# Mlh1–Pms1 couples ATP-driven DNA compaction with nick-dependent endonuclease activation

**DOI:** 10.1093/nar/gkaf1252

**Published:** 2025-12-03

**Authors:** Bryce W Collingwood, Amruta N Bhalkar, Carol M Manhart

**Affiliations:** Department of Chemistry, Temple University, Philadelphia, PA 19122, United States; Department of Chemistry, Temple University, Philadelphia, PA 19122, United States; Department of Chemistry, Temple University, Philadelphia, PA 19122, United States

## Abstract

In eukaryotes, mismatch repair begins with MutS homolog (MSH) complexes detecting mismatches and recruiting the proliferating cell nuclear antigen (PCNA)-stimulated endonuclease Mlh1–Pms1/PMS2 (yeast/human), which nicks the DNA to allow downstream proteins to remove the mismatch. Although Mlh1–Pms1 is an ATPase and this activity is essential *in vivo*, ATP is not required for nicking *in vitro*, leaving its function unresolved. Using yeast proteins, we show that Mlh1–Pms1 uses ATP to compact continuous DNA, a behavior which may act as a search mechanism for strand-discrimination signals. When a pre-existing nick is encountered, compaction is suppressed and Mlh1–Pms1 instead stabilizes the site, protecting it from replication factor C (RFC)/PCNA-induced melting. Phased nicking assays further reveal that the timing of Mlh1–Pms1 encountering a pre-existing nick relative to RFC/PCNA determines whether the complex remains suppressed or becomes activated. Together, these results support a model of Mlh1–Pms1 using ATP-driven global compaction to toggle between a search mode and a PCNA-licensed repair mode.

## Graphical Abstract

**Graphical Abstract:**
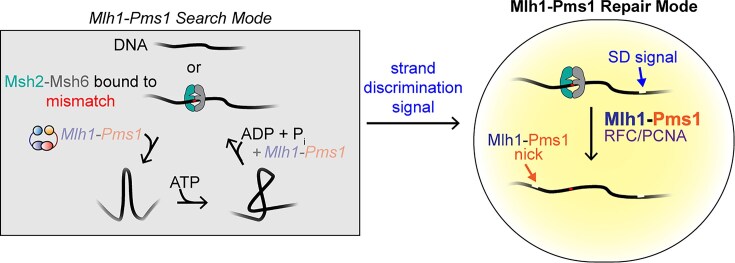

## Introduction

DNA mismatch repair is a highly conserved biological process crucial for maintaining genomic stability. It primarily corrects mispaired nucleotides that arise from errors during DNA replication or repair, thereby preventing the accumulation of mutations. Defects in this process are associated with Lynch syndrome, a hereditary cancer syndrome characterized by increased cancer risk due to unrepaired DNA mutations [1]. In eukaryotic mismatch repair, mismatches are recognized by either the Msh2–Msh6 or Msh2–Msh3 complex, which have overlapping specificities [2]. These MutS homolog (MSH) complexes recruit the MutL homolog endonuclease (Mlh1–Pms1 in yeast; MLH1–PMS2 in humans and mice). The Mlh1–Pms1/PMS2 complex is stimulated by the replication processivity clamp PCNA to nick one strand of the DNA duplex [3]. This nick serves as an entry point for other factors that remove the mismatch through polymerase strand displacement and flap cleavage, or through excision and resynthesis [3, 4].

The Mlh1–Pms1/PMS2 complex is critical for initiating mismatch removal in eukaryotic systems. This protein consists of two homologous subunits, each containing a globular amino-terminal domain important for DNA binding and ATPase activity [5–8]. These domains are connected by intrinsically disordered regions to globular carboxy-terminal domains, which contain the primary dimerization site between the Mlh1 and Pms1/PMS2 subunits. The endonuclease active site is primarily located in the carboxy-terminal domain of the Pms1/PMS2 subunit [9].

In addition to this catalytic site, Mlh1–Pms1/PMS2 also interacts with PCNA, predominantly through the carboxy-terminal domain of the Pms1/PMS2 subunit. This interaction stimulates endonuclease activity and serves as a licensing step for incision [10–12]. PCNA has further been shown to bias activity toward the DNA strand containing a pre-existing nick *in vitro*, where the nick is hypothesized to act as a loading site for PCNA and orients its interaction face to direct Mlh1–Pms1/PMS2 incision to one strand of the duplex [10, 13, 14]. How exactly strand discrimination is achieved *in vivo* is unknown, but may involve replication-associated strand breaks, such as those generated during Okazaki fragment processing or spontaneous events [15]. Recent work further suggests that yeast Mlh1–Pms1 may preserve these strand breaks to ensure they are available for downstream mismatch repair, but a precise mechanism for how Mlh1–Pms1/PMS2 carries out this function or for what downstream purpose has not been described [16].

The Mlh1–Pms1/PMS2 complex is highly dynamic and undergoes significant conformational changes mediated by its intrinsically disordered regions [17–23]. Studies on the *Escherichia coli* MutL homodimer, which is homologous to Mlh1–Pms1/PMS2, suggest that ATP binding induces conformational changes that allow the amino-terminal domains to interact like a clamp, with ATP mediating the opening and closing of the protein’s structure [17, 18]. Similar dynamics have been observed in yeast and human systems, where asymmetric ATPase activities in the Mlh1 and Pms1/PMS2 subunits drive sequential and allosteric conformational changes [19, 24–27]. Visual evidence from atomic force microscopy studies in yeast and human systems shows that ATP binding leads to large-scale structure changes, with the intrinsically disordered regions condensing to bring the amino- and carboxy-terminal domains closer together [19]. However, these conformational changes have primarily been studied in the absence of DNA, leaving their role for DNA-bound states unclear.

ATPase activity not only controls the conformational dynamics of Mlh1–Pms1/PMS2 but is also critical for its *in vivo* function. In yeast, mutations that disrupt ATP binding in either the Mlh1 or Pms1 subunits result in mutation rates similar to those observed in *mlh1Δ* or *pms1Δ* strains, demonstrating the importance of ATPase activity in mismatch repair [24, 25]. Yet, both yeast Mlh1–Pms1 and nucleolytic MutL from *B. subtilis* can nick DNA without ATP *in vitro*, suggesting that ATPase activity is not required for incision itself. Instead, ATP is thought to drive conformational changes in Mlh1–Pms1 which may allow the protein to coordinate with other factors before or after nicking [20, 28, 29]. Biochemical studies support this idea, showing ATP-driven rearrangements of the intrinsically disordered regions that may influence how the complex engages DNA [20, 21, 23]. Despite these insights, the precise mechanisms by which ATPase activity regulates Mlh1–Pms1/PMS2’s functions in mismatch repair and its interactions with DNA remain to be elucidated.

In addition to its dynamic nature, Mlh1–Pms1/PMS2 binds DNA cooperatively, with multiple copies assembling on DNA during mismatch repair [30–36]. A role for an Mlh1–Pms1/PMS2 oligomer in DNA mismatch repair has not been clearly established, but may provide an explanation for observations that Mlh1–Pms1/PMS2 nicks have been observed in some systems at distances on the order of a few hundred nucleotides from the mismatch [10, 11]. If Mlh1–Pms1/PMS2 nicks distant to the mismatch, multiple copies of Mlh1–Pms1/PMS2 may serve as a communication channel between Msh2–Msh6 bound to the mismatch and the nick site. This is consistent with recent work suggesting that MutL homolog complexes can restrain Msh2–Msh6 at the mismatch, although other models suggest that MutS and MutL may move together [33, 37–40].

Beyond acting as a physical bridge, Mlh1–Pms1/PMS2 oligomers have been proposed to promote DNA–DNA interactions. Although previously suggested for the meiotic homolog Mlh1–Mlh3 using *Saccharomyces cerevisiae* as a model organism [34], yeast Mlh1–Pms1 complexes have been shown to simultaneously interact with two distinct DNA substrates and that this tethering feature can stimulate Mlh1–Pms1 endonuclease activity [35]. These data are consistent with atomic force microscopy work, visually suggesting that tracts of Mlh1–Pms1/PMS2 can form on DNA and alter the shape of the substrate [30, 33]. How Mlh1–Pms1/PMS2 changes the shape of the DNA in mismatch repair and whether large-scale conformational changes in Mlh1–Pms1/PMS2 mediated by ATP control Mlh1–Pms1/PMS2 complexes and DNA–DNA associations and conformation remains to be determined.

In this study, using purified Mlh1–Pms1 from *S. cerevisiae*, we observed that Mlh1–Pms1 promotes DNA conformational changes *in vitro*, which are modulated by ATP. We also found that sequence features affecting DNA conformation interfere with Mlh1–Pms1’s ability to promote DNA remodeling, potentially explaining instability in genomic regions containing these sequences. Additionally, we found that pre-existing DNA nicks suppress Mlh1–Pms1’s ability to compact DNA. In contrast, when Mlh1–Pms1 compacts DNA prior to encountering a pre-existing nick, endonuclease activity is promoted.

## Materials and methods

### Reagents used in this study

Wild-type Mlh1-FLAG-Pms1 was used for all experiments unless specifically noted. Mlh1-FLAG-Pms1 was expressed from a strain transformed with pMH8, which was a gift from Thomas Kunkel’s lab and pEAE269, which was a gift from Eric Alani’s lab [23, 41]. pEAE269 is pMH1, originally constructed by Thomas Kunkel’s lab, with a FLAG tag inserted at amino acid 499 of Mlh1. The Mlh1–Pms1 ATPase mutants (mlh1N35A-pms1N34A, mlh1N35A-Pms1, and Mlh1-pms1N34A) were generated by Q5 site-directed mutagenesis (NEB) using pMH1 and pMH8 as described previously [29]. Yeast RFC, PCNA, and Msh2–Msh6 were expressed and purified as previously reported [42–44].

Unless otherwise indicated, supercoiled plasmid substrates were pUC18 or pUC19 and relaxed plasmid substrates were topoisomerase relaxed pUC18 or pUC19. pUC18 and pUC19 are identical in size and sequence and only differ by the direction of the multiple cloning site. The relaxed forms were generated by incubating 3.36 pmol of supercoiled pUC18/pUC19 with 30 units of DNA Topoisomerase I (Topo I; NEB) for 1 h at 37°C in a 60 μl reaction containing 50 mM potassium acetate, 20 mM Tris–acetate, 10 mM magnesium acetate, 100 μg/ml recombinant albumin, pH 7.9). The topoisomerase was then inactivated by incubating at 80°C for 20 min.

Plasmid substrates containing a non-B-form, nucleotide repeat segment were generated by digesting 3.36 pmol of pUC18 with EcoRI (NEB) and BamHI (NEB). The linear fragment was gel extracted (QIAGEN) and 1.4 nM of the extracted product was then incubated with 2 μM of annealed complementary oligonucleotides (Supplementary Table S1). Four hundred units of T4 DNA ligase (NEB) was included and incubated for 30 min at 16°C in a final volume of 20 μl. The DNA was then transformed and propagated using DH5α cells, miniprepped, and insertion of the desired sequence was confirmed by Sanger sequencing (Azenta).

The formation of non-B-form structures was verified using T7 endonuclease I, a structure-selective endonuclease that acts on non-B-form DNA. To do this, 3.8 nM of miniprepped, supercoiled plasmid was incubated with 100 units of T7 endonuclease I (NEB) in a reaction containing 10 mM Tris–HCl, 50 mM NaCl, 10 mM MgCl_2_, 1 mM dithiothreitol (DTT), at pH 7.9 for 45 min at 37°C. Reaction products were analyzed using a 1% agarose gel stained with 1.5 μg/ml ethidium bromide. To characterize the linearized forms of the substrates, an identical procedure was performed immediately after linearization with BsaI-Hfv2 (NEB), which was performed at 37°C for 60 min, followed by heat inactivation of the enzyme according to the manufacturer’s instructions.

Heteroduplex plasmid substrates containing a single GT mispair were generated by a previously reported method [45]. Briefly, 18 μg of pUC19 was digested with 70 units of Nt.BspQI (NEB) for 60 min at 37°C in buffer containing 50 mM potassium acetate, 20 mM Tris–acetate, pH 7.9, and 10 mM magnesium acetate. The reaction was then supplemented with 40 units of T7 exonuclease (NEB) and incubated for an additional 60 min at 37°C to excise a single strand of the plasmid. A phosphorylated oligonucleotide containing the mismatch (CMO487; Supplementary Table S1) was annealed at a 4:1 molar ratio (oligonucleotide:single-stranded plasmid), by incubating at 95°C for 5 min followed by gradual cooling for 120 min. Thirty units of T4 DNA polymerase (NEB), 2000 units of T4 DNA ligase (NEB), 1 mM dNTPs, 10 mM MgCl_2_, and 1 mM ATP, were then added and incubated at 37°C for 120 min. To remove residual template and unannealed product, the reaction was treated with 20 units of T5 exonuclease for 60 min at 37°C. Mismatch-containing plasmid was purified using a MinElute Reaction Cleanup Kit (Qiagen). Aliquots were analyzed on a 1% agarose gel in 1× TAE [40 mM Tris–acetate, 1 mM ethylenediaminetetraacetic acid (EDTA)] containing 0.66 μg/ml ethidium bromide to verify DNA processing at each step. For substrates requiring a pre-existing nick, plasmids were additionally nicked with Nt.BspQI (NEB) following the manufacturer’s instructions, then heat inactivated.

### Mismatch localization assays

To assess the localization of mismatch repair factors on plasmid substrates, a method similar to one used previously was adapted [46]. Seventy-six femtomoles of heteroduplex, nicked heteroduplex, or nicked homoduplex were incubated with 50 nM Msh2–Msh6 and/or 50 nM Mlh1–Pms1 for 3 min at 25°C in a buffer containing 20 mM Tris acetate, pH 7.9, 10 mM magnesium acetate, 50 mM potassium acetate, 50 mM NaCl, 100 μg/ml recombinant bovine serum albumin (BSA) (NEB), 2 mM MgCl_2_, and 0.5 mM ATP. Immediately afterward, 0.2 units of a designated restriction enzyme (HindIII-HF, EcoRI-HF, NdeI, or XmnI; NEB) were added, mixed, and incubated for 10 min at 37°C. Reactions were terminated by boiling at 95°C for 5 min to denature DNA-bound proteins. Samples were then resolved on 1% agarose gels containing 0.66 μg/ml ethidium bromide in 1× TAE.

### High-throughput DNA binding assays for measuring affinity for different DNA topologies

A pool of topologically distinct, DNA isomers was generated by incubating 3.36 pmol of pUC19 plasmid with 5 units of DNA Topo I (NEB) for 1 min at ambient room temperature in the supplied 1× CutSmart Buffer (NEB), followed by heat inactivation of the enzyme at 80°C for 20 min. Twenty microliter reactions were assembled in Buffer A, containing 20 mM HEPES–KOH, pH 7.5, 6% glycerol, 200 μg/ml bovine serum albumin (BSA), 2 mM MgCl_2_, and 1 mM DTT, along with 76 fmol (total DNA) of the topoisomer pool, and 200 nM Mlh1-FLAG-Pms1 (final concentrations). Reactions were incubated for 30 min at 4°C and oscillated at 20 revolutions per minute (RPM). Mlh1-FLAG-Pms1 and any associated DNA molecules were immunoprecipitated using M2 anti-FLAG magnetic beads (Pierce). Briefly, 30 μl of suspended beads (25% slurry in phosphate buffered saline, pH 7.2, containing 0.01% Tween-20 detergent and 0.02% sodium azide) were added to each reaction and incubated for 30 min at 4°C with constant oscillation at 20 RPM. The supernatant was then separated from the beads using neodymium magnets and removed. The beads were washed with 10 μl of Buffer A three times and resuspended in 20 μl of Buffer A containing 0.96 units of proteinase K. The material was then incubated at 37°C for 30 min with constant oscillation at 20 RPM followed by incubation at 95°C for 5 min to fully denature and degrade Mlh1-FLAG-Pms1 and dissociate the DNA from the beads. The denatured supernatant was then analyzed by 1% (w/v) agarose gel resolved in 1× TBE (89 mM Tris base, 89 mM boric acid, and 2 mM EDTA) and stained in 1× TBE containing 1× GelRed (Biotium). Methods for quantifications are described in the Results section as well as in the supplemental information.

### DNA topoisomerase and gyrase enhancement assays

Mlh1–Pms1’s ability to stimulate *E. coli* Topo I (NEB) and *E. coli* DNA Gyrase (Sigma) were assayed by similar methods. For the Topo I enhancement assays, 76 fmol of supercoiled pUC18 was incubated with increasing concentrations of Mlh1–Pms1 (50, 100, or 200 nM) for 10 min at 25°C in a buffer containing 50 mM potassium acetate, 20 mM Tris–acetate, pH 7.9, 10 mM magnesium acetate, and 100 μg/ml BSA in a final volume of 20 μl. After the initial incubation, 0.05 units of Topo I (NEB) were added, and the reactions were incubated for 20 min at 37°C. Reactions were stopped with 0.96 units of proteinase K, 1% sodium dodecyl sulphate (SDS), and 14 mM EDTA (final concentrations), then resolved by a 1% (w/v) agarose gel, electrophoresed in 1× TBE for 75 min. Gels were stained in 1× TBE containing 1.6 μg/ml ethidium bromide for 30 min. For the Gyrase enhancement assays, pre-relaxed pUC18 was incubated for 10 min at 25°C in a 20 μl reaction with the same concentrations of Mlh1–Pms1 above in a buffer containing 35 mM Tris–HCl, pH 7.5, 24 mM KCl, 4 mM MgCl_2_, 1 mM ATP, 2 mM DTT, 1.8 mM spermidine, 6.5% glycerol, 100 μl/ml BSA. After this initial incubation, 0.0075 units of *E. coli* DNA Gyrase were added, and the reactions were incubated for 30 min at 37°C. The enhancement of both enzymes was quantified by comparing the band intensities of either fully relaxed DNA, in the case of Topo I, or fully supercoiled DNA, in the case of Gyrase, relative to the input DNA concentrations for the respective set of reactions.

### Ultraviolet-based structural probing assay

For assays measuring protein-induced DNA compaction and RFC-induced DNA melting, 76 fmol of supercoiled pUC18, relaxed pUC18, 3′-nicked heteroduplex, or nicked homoduplex was incubated with Mlh1–Pms1, Msh2–Msh6, RFC, or PCNA as indicated in Buffer A containing 150 mM NaCl. A final concentration of 0.5 mM ATP was included where indicated. Reactions were irradiated with ultraviolet (UV) light (300 nm) for 60 min at room temperature in 200 μl transparent PCR tubes (VWR #76318-804), 15 cm from the light source in a Rayonet Photochemical Reactor-200 equipped with 12 lamps outputting 8 W each (Southern New England Ultraviolet Company RPR-3000A). Following irradiation, reactions were treated with 0.96 units of proteinase K for 15 min at 37°C, and products were analyzed by 1% (w/v) agarose gel containing 0.66 μg/ml ethidium bromide and resolved in 1× TAE (40 mM Tris–acetate, 1 mM EDTA).

### T7 endonuclease I enhancement assays

Similar procedures were used to test Mlh1–Pms1’s ability to stimulate a structure-specific endonuclease on both supercoiled and nicked pUC18. The nicked pUC18 was generated by incubating supercoiled plasmid with Nt.BspQI (NEB) according to the manufacturer’s instructions, followed by heat inactivation. To measure T7 endonuclease I stimulation, DNA (3.8 nM) was added to a 20 μl reaction containing 50 mM NaCl, 10 mM Tris–HCl, pH 7.9, 10 mM MgCl_2_, and 1 mM DTT (final concentrations). Where indicated, 50 nM Mlh1–Pms1 was added to reactions containing supercoiled pUC18, and 100 nM Mlh1–Pms1 was added to reactions containing nicked pUC18. Reactions were incubated at room temperature for 10 min to allow Mlh1–Pms1 to bind to the DNA. Subsequently, 0.5 mM ATP or ATPγS was added where indicated, and the reactions were incubated at 37°C for 15 min. Following this, 0.2 units of T7 endonuclease I (NEB) was added to reactions with supercoiled pUC18, and 0.6 units were added to reactions with nicked pUC18, as indicated. The reactions were incubated at 37°C for 45 min and stopped by adding 1% SDS, 14 mM EDTA, and 0.96 units of proteinase K (final concentrations). The reaction products were resolved on a 1% (w/v) agarose gel containing 0.66 μg/ml ethidium bromide in 1× TAE buffer.

### ATPase assays

ATPase activity was measured using thin layer chromatography as previously reported [12, 24]. Briefly, 10 μl reactions were prepared in a buffer containing 20 mM HEPES–KOH, pH 7.5, 20 mM KCl, 1% glycerol, 2 mM MgCl_2_, 2.5 mM MnSO_4_, and 40 μg/ml BSA including 3.8 nM of either supercoiled pBR322 or pBR322 relaxed with a topoisomerase as described above, 104 μM [γ-^32^P]-ATP (Perkin Elmer), and 400 nM of Mlh1–Pms1 (final concentration). Reactions were incubated for 45 min at 37°C. ATP hydrolysis products were analyzed using polyethylenimine cellulose plates resolved in a solution of 0.8 M LiCl and 1 M formic acid for 35 min. Plates were phosphor-imaged using a Sapphire Biomolecular Imager (Azure). To quantify the amount of ATP hydrolysis, the density of the spot corresponding to hydrolysis product was quantified relative to the total amount of signal in each lane. The proportion of [γ-^32^P]-ATP hydrolyzed was then converted to pmol of ATP using the total number of pmol of ATP in the reaction.

### Electrophoretic mobility shift assays

Electrophoretic mobility shift assays were used to assess DNA binding to plasmid-based substrates. Supercoiled or relaxed pUC18 DNA (76 pmol) or its derivative with a dinucleotide repeat insert was incubated with increasing concentrations of Mlh1–Pms1 in 20 μl reactions in a buffer containing 20 mM HEPES–KOH, pH 7.5, 6% glycerol, 2 mM MgCl_2_, 1 mM DTT, and 200 μg/ml BSA for 20 min at ambient room temperature, unless otherwise indicated. Mlh1–Pms1-bound DNA was separated from unbound DNA using a 1% (w/v) agarose gel resolved in 1× TAE on ice and stained with 1.5 μg/ml ethidium bromide.

### Endonuclease assays

Endonuclease assays were performed as previously described [20, 34, 35, 47]. Briefly, 76 pmol of DNA substrate was incubated with the indicated concentrations of Mlh1–Pms1 in 20 μl reactions containing 20 mM HEPES–KOH, pH 7.5, 20 mM KCl, 1% glycerol, 2.5 mM MnSO_4_, 0.5 mM ATP, and 200 μg/ml BSA. Final concentrations of PCNA and RFC were 0.5 μM and 0.1 μM, respectively. Reactions were incubated at 37°C for 60 min, followed by the addition of 0.96 units of proteinase K, 1% SDS, and 14 mM EDTA (final concentrations) to stop the reaction. Endonuclease products were analyzed by either a 1% (w/v) agarose gel containing 0.66 μg/ml ethidium bromide for supercoiled substrates or a 1% (w/v) agarose gel containing 30 mM NaCl and 2 mM EDTA when a denaturing agarose gel was used to measure nicking on relaxed or linear DNA topologies. Denaturing gels were resolved in a buffer solution containing 30 mM NaOH and 2 mM EDTA at 30 V for 2.5 h, followed by neutralization in a 1 M Tris–HCl, pH 7.5 buffer and staining with 0.5 μg/ml ethidium bromide.

To quantify the amount of supercoiled DNA nicked by Mlh1–Pms1 using native agarose gel analysis, band intensities corresponding to the supercoiled starting material and the nicked product were measured. The proportion of nicked product was calculated relative to the sum of these bands, subtracting the DNA signal at the relaxed or nicked position in the negative control lanes. To quantify the amount of relaxed or linear DNA nicked by Mlh1–Pms1 using denaturing agarose gel analysis, band intensities of the starting material were measured, and the signal loss relative to the starting material in negative controls was calculated.

### Gel imaging

Gels were imaged on a Sapphire Biomolecular Imager (Azure) quantified using ImageJ software (NIH). Quantifications for individual assays are described under each assay. Where gel images are present, representative images are shown. Quantifications below gels refer to the selected image, averages between replicates, and standard deviations between replicates.

## Results

### Mismatch and nick-dependent recruitment of Mlh1–Pms1 and Msh2–Msh6

**Figure 1.**
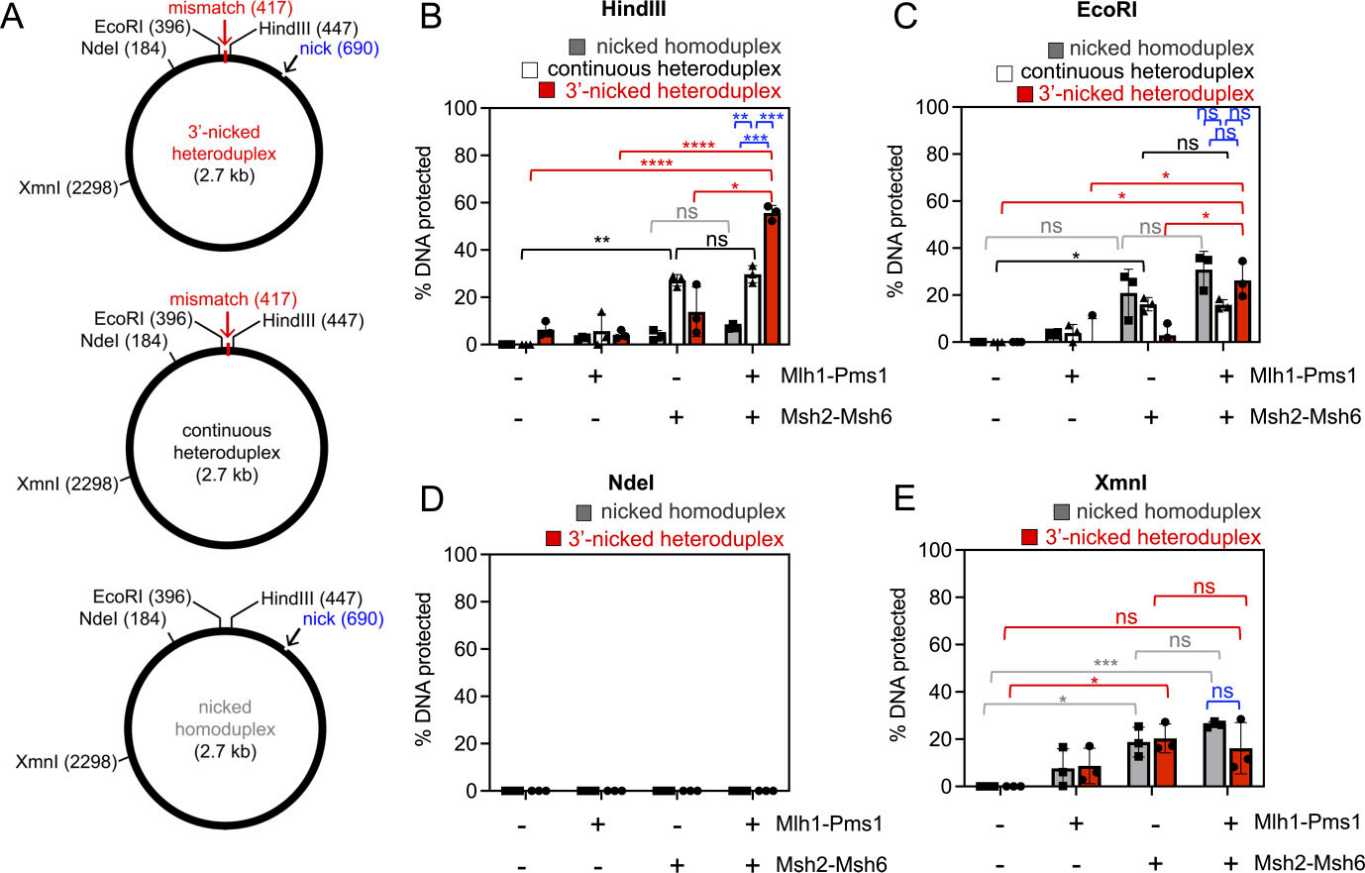
Mlh1–Pms1 and Msh2–Msh6 localize near a mismatch when a nick is present in the substrate. (**A**) Schematics of pUC19-based substrates used to assay protection from restriction enzyme digestion, with positions of restriction sites indicated. Where present, a pre-existing nick was introduced using Nt.BspQI. Numbers in parentheses indicate nucleotide positions. (**B–E**) Quantification of restriction digests shown in Supplementary Fig. S1. Reactions contained 50 nM Mlh1–Pms1 and 50 nM Msh2–Msh6, as indicated, prior to addition of the restriction enzyme as described in the ‘Materials and methods’ section. All reactions were performed with 100 mM total salt. Experimental lanes were normalized to negative controls lacking mismatch repair proteins. Bars represent means, with error bars representing standard deviations between experiments. Statistical significance was determined using unpaired *t*-tests with Welch’s correction (Prism 10). Significance is denoted above bars (*P <.05; **P <.005; ***P <.0005; ****P <.0001; ns, not significant). A representative list of *P*-values is provided in Supplementary Table S2.

To establish conditions that direct Mlh1–Pms1 to specific sites during DNA mismatch repair, we first tested how mismatches and pre-existing nicks that may act as strand-discrimination signals affect its recruitment. To do this, we generated a plasmid substrate similar to those previously used to measure strand-specific nicking by Mlh1–Pms1 [10, 11, 13, 14, 48]. The plasmid contains a GT mispair between the EcoRI and HindIII sites and a pre-existing nick ∼270 bp downstream (3′) of the mismatch (Fig. 1A). To determine whether the mismatch or nick influences the localization of repair factors, we incubated substrates with yeast Msh2–Msh6 and/or Mlh1–Pms1 and monitored cleavage by restriction enzymes adjacent to the mismatch. If proteins occupy DNA near the mismatch, EcoRI or HindIII cleavage should be at least partially inhibited [46], similar to DNase I footprinting principles.

At the HindIII site, digestion was efficient in the presence of either Msh2–Msh6 or Mlh1–Pms1 or in combination when a nicked homoduplex was used, indicating little or no protection and suggesting that neither factor localized to the probed region on this substrate (Fig. 1B, gray bars, and Supplementary Fig. S1D and E). With a mismatch present, however, HindIII cleavage was inhibited when both proteins were included (Fig. 1B, red bars, and Supplementary Fig. S1C and E), whereas either protein alone provided minimal protection. For heteroduplex DNA lacking a nick, Msh2–Msh6 conferred modest protection, but Mlh1–Pms1 did not enhance this effect as it did on nicked heteroduplex DNA (Fig. 1B, compare white versus red bars, and Supplementary Fig. S1F and J).

At the EcoRI site, Mlh1–Pms1 alone had no effect, but Msh2–Msh6 protected against digestion on the nicked homoduplex and continuous heteroduplex. This effect is likely due to enhanced local DNA flexibility, as predicted by DNAcycP2 (Supplementary Fig. S1B) [49–52]. When both a mismatch and a nick were present, Msh2–Msh6 no longer protected the site efficiently, likely because the proteins were titrated to the nick or mismatch and were two competing binding sites that won over flexibility. Notably, when both proteins were present together, the EcoRI site was protected in the 3′-nicked heteroduplex substrate relative to the presence of either protein alone (Fig. 1C and Supplementary Fig. S1G–J). When comparing among substrates in the presence of both repair proteins, protection of this site is independent of the mismatch, but dependent on the nick, suggesting the importance of this DNA feature in localizing the mismatch repair factors adjacent to the mismatch.

At the more distal NdeI site, located on the same side of the mismatch as EcoRI, no protection was observed under any condition (Fig. 1D and Supplementary Fig. S1K–M), confirming that localization is restricted to the mismatch region. At the even further XmnI site, Msh2–Msh6 provided slight protection (Fig. 1E and Supplementary Fig. S1O–Q), but this was mismatch-independent and again likely due to sequence flexibility. Mlh1–Pms1 did not enhance protection, indicating that mismatch repair factors are not stabilized at distal sites but rather concentrate near the mismatch.

### Mlh1–Pms1 reshapes DNA and is regulated by ATP binding and pre-existing nicks

**Figure 2.**
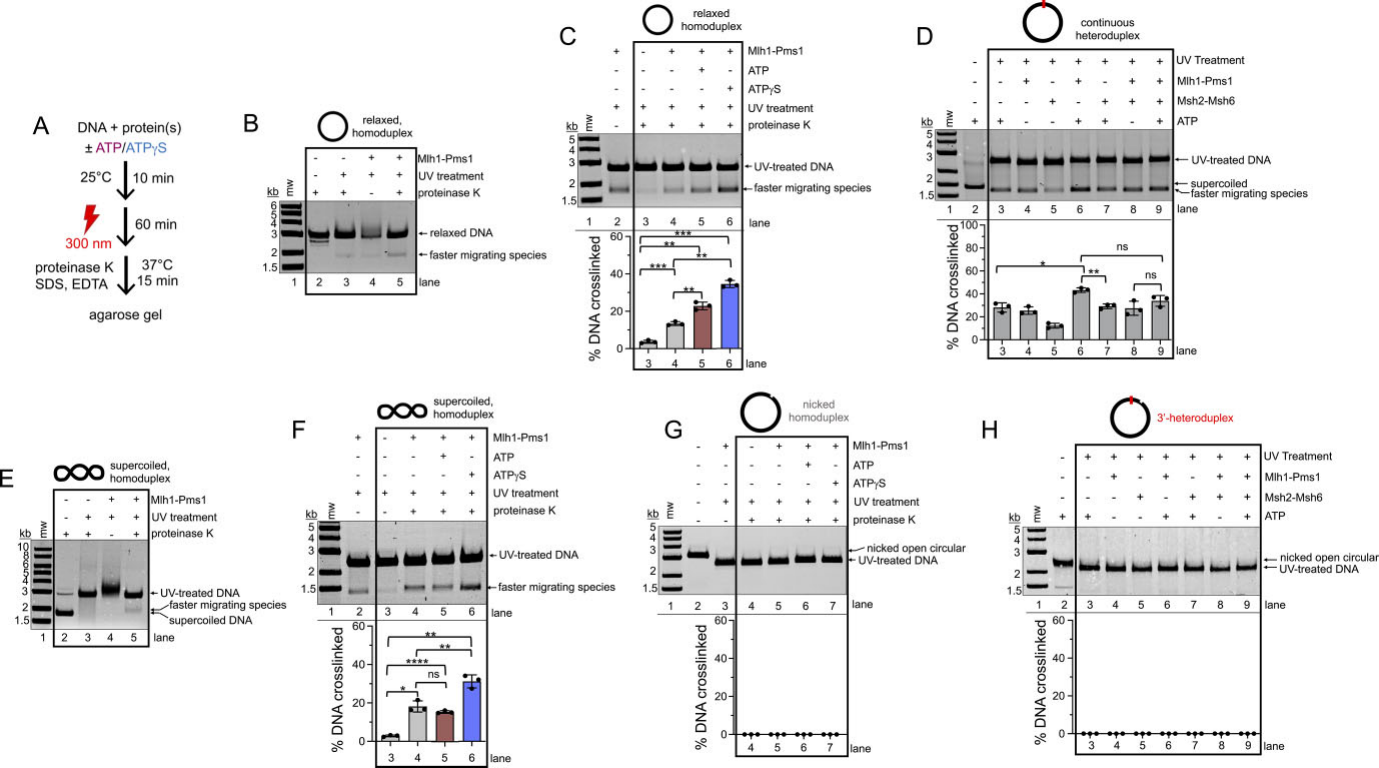
Mlh1–Pms1 rearranges DNA in an ATP-dependent mechanism and is inhibited by a pre-existing nick. (**A**) Schematic for UV-based structural probing assay used to detect Mlh1–Pms1-dependent DNA–DNA crosslinks. Plasmid substrates were incubated with Mlh1–Pms1 in the presence or absence of ATP or ATPγS. Reactions were then irradiated, deproteinated, and analyzed by agarose gel. Quantifications reflect the percentage of faster migrating species relative to the total amount of DNA in each lane (see the ‘Materials and methods’ section for additional details). (**B**) Control experiment showing the effect of UV treatment on relaxed DNA with and without Mlh1–Pms1 (100 nM) in reactions containing 20 mM NaCl and proteinase K. (**C, D**) Effect of nucleotide on formation of the faster migrating DNA species captured by UV-crosslinking on relaxed homoduplex or relaxed heteroduplex. Where present, Mlh1–Pms1 is included at 100 nM, Msh2–Msh6 is included at a final concentration of 50 nM, and ATP or ATPγS was included at a final concentration of 0.5 mM in a buffer containing 150 mM NaCl. All lanes in panel (D) were treated with proteinase K. (**E**) Control experiment showing the effect of UV treatment on supercoiled DNA with and without Mlh1–Pms1 (100 nM) in reactions containing 20 mM NaCl and proteinase K. (**F–H**) Effect of nucleotide and a pre-existing nick on formation of the faster migrating DNA species captured by UV-crosslinking on supercoiled and homoduplex and heteroduplex containing a pre-existing nick as shown in Fig. 1A. Reactions were performed as in panels (C) and (D). All lanes in panel (H) were treated with proteinase K. For all panels showing quantifications, bar graphs represent means with error bars representing standard deviations between experiments. Statistical significance was determined using unpaired *t*-tests with Welch’s correction (Prism 10). Significance is denoted above bars (*P <.05; **P <.005; ***P <.0005; ****P <.0001; ns, not significant).

Since Mlh1–Pms1 recruitment depends on both mismatch recognition by Msh2–Msh6 and the presence of a nick, we next asked how the complex engages DNA once it is localized. To test whether Mlh1–Pms1 complexes alter global DNA structure, we used a UV-crosslinking assay that reports on protein-induced DNA compaction. We performed this assay under conditions similar to those in the localization assay, but to establish a baseline in the absence of a mismatch, we first examined relaxed homoduplex plasmids. In this assay, purified Mlh1–Pms1 was incubated with relaxed homoduplex plasmid DNA, and reactions were performed with or without ATP or the nonhydrolyzable analog ATPγS. UV exposure at 300 nm induces inter- and intra-strand crosslinks, creating a covalent attachment between DNA regions brought into close proximity transiently by Mlh1–Pms1 binding, allowing us to take a snapshot of potential DNA reshaping, such as compaction or loop formation (Fig. 2A and B).

Using relaxed homoduplex in this assay, we observed that addition of Mlh1–Pms1 resulted in a shift to a more heterogeneous DNA population, likely reflecting Mlh1–Pms1 binding or crosslinking (Fig. 2B, compare lane 4 with lanes 2 and 3). This shift was not the result of Mlh1–Pms1 nicking the DNA, because this assay was performed under conditions where Mlh1–Pms1’s endonuclease activity is not observed due to the absence of RFC or PCNA (Supplementary Fig. S2A and B). After proteinase K treatment to degrade the protein component, a new faster-migrating DNA species appeared (Fig. 2B, lane 5). A small amount of this species was observed without Mlh1–Pms1 (Fig. 2B, lane 3), possibly due to the plasmid’s natural conformational flexibility, but its abundance increased markedly with Mlh1–Pms1 and proteinase K. This suggests that Mlh1–Pms1 may promote intra-molecular DNA–DNA associations, generating a looped or more compact DNA structure.

We hypothesized Mlh1–Pms1 uses ATP to alter DNA shape, because ATP has been shown to induce large conformational changes in Mlh1–Pms1 [17–19, 21]. To test this, we performed the assay as shown in Fig. 2B at different ionic strengths and with varying nucleotide conditions. We observed that at both 20 mM NaCl and near-physiological 150 mM NaCl, inclusion of ATP and ATPγS promoted the formation of the Mlh1–Pms1-dependent faster migrating DNA species (Supplementary Fig. S2C and Fig. 2C). We also performed the assay on relaxed, continuous heteroduplex DNA that was used in Fig. 1 to promote localization of mismatch repair factors. Using this substrate at 150 mM NaCl, we found that although Mlh1–Pms1 still promoted formation of the faster migrating species in an ATP-stimulated manner (Fig. 2D, compare lanes 6–3 and 4), inclusion of Msh2–Msh6 did not significantly affect this behavior (Fig. 2D, compare lanes 6 and 9), suggesting that DNA compaction by Mlh1–Pms1 may be independent of recruitment by Msh2–Msh6.

Using supercoiled plasmid DNA in this assay, we observed that UV exposure alone altered DNA migration, likely due to strand breaks (Fig. 2E) [53, 54]. Similar to relaxed plasmid assays, adding Mlh1–Pms1 to the reaction created a more heterogeneous population, and proteinase K treatment revealed a faster-migrating DNA species, consistent with compacted DNA (Fig. 2E, lane 5). Resembling that of relaxed substrates, this product formed at both low and physiological salt (Supplementary Fig. S2D and Fig. 2F). Unlike relaxed DNA, however, ATP did not significantly stimulate formation of this species at physiological salt, and at low salt ATP even reduced product formation relative to the no-nucleotide condition. By contrast, ATPγS consistently enhanced formation of the faster-migrating species under both conditions (Fig. 2F, compare lanes 4 and 6, and Supplementary Fig. S2D). These results suggest that Mlh1–Pms1 may use ATP differently on relaxed versus supercoiled DNA.

When a pre-existing nick was introduced into either relaxed homoduplex or heteroduplex substrates, the faster-migrating species was not detected under any nucleotide condition (Fig. 2G and H). Notably, the substrate used in Fig. 2H is identical to that in Fig. 1, and the assays were performed under comparable conditions, where a mismatch, Msh2–Msh6, and a pre-existing nick promoted protection of restriction sites adjacent to the mismatch. Taken together, these findings suggest that ATP promotes Mlh1–Pms1–dependent compaction on continuous DNA, whereas a pre-existing nick prevents compaction, and may represent a distinct mode.

### ATP modulates Mlh1–Pms1 activities on supercoiled DNA distinctly from relaxed DNA

Previous studies have suggested an interplay between Mlh1–Pms1’s ATPase activity and DNA binding, with DNA stimulating ATPase activity and ATP increasing dissociation constants for small oligonucleotide substrates [17, 20, 23]. Since we observed a different effect of ATP on formation of the faster migrating DNA band in assays using supercoiled versus relaxed plasmid substrates, we investigated whether Mlh1–Pms1’s affinity for different DNA topologies is affected by ATP. Using an electrophoretic mobility shift assay, we measured Mlh1–Pms1’s apparent DNA-binding affinity in the presence of ATP or ATPγS compared to conditions without nucleotide.

**Figure 3.**
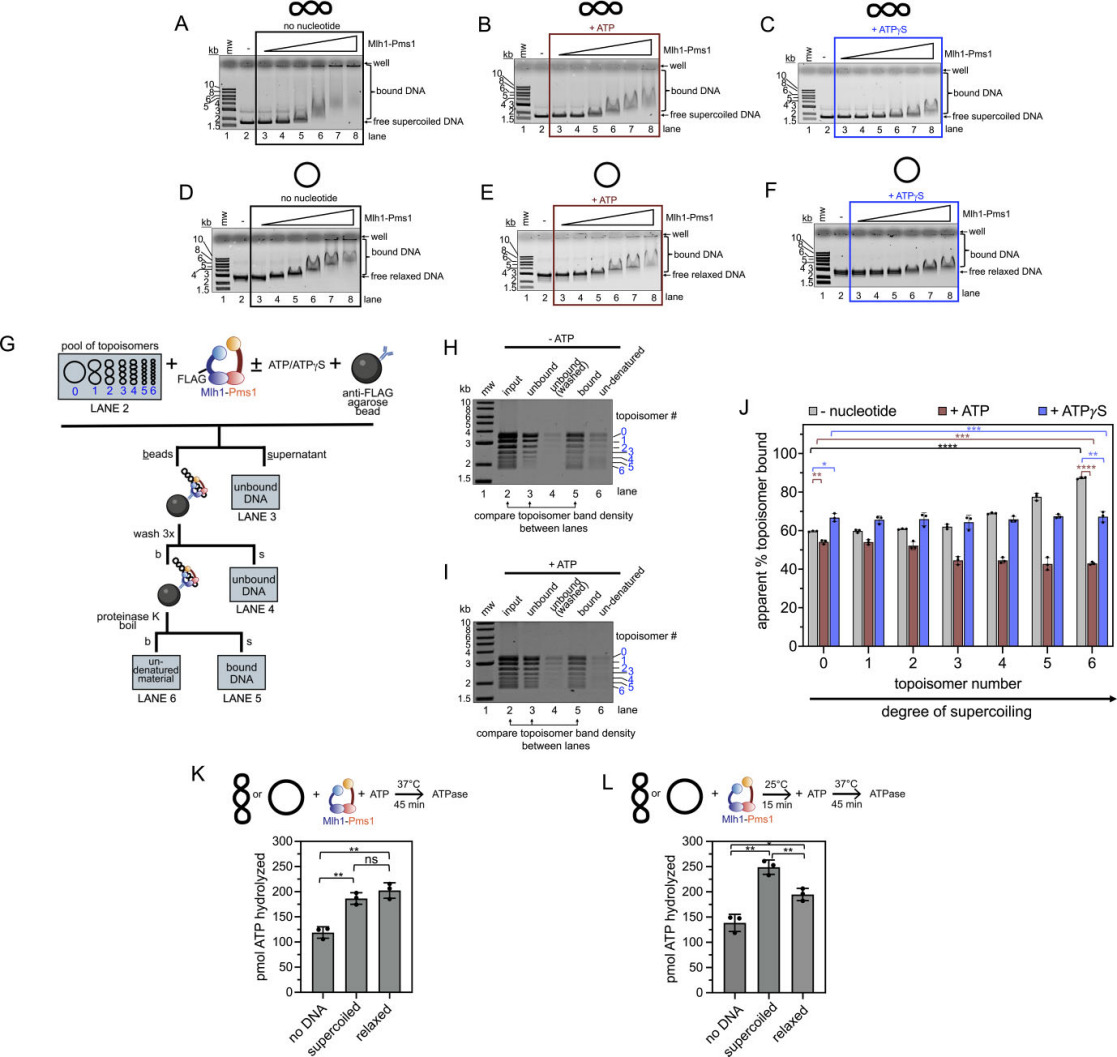
Mlh1–Pms1 is more sensitive to ATP on supercoiled DNA than relaxed topologies. (**A–F**) Electrophoretic mobility shift assays using either supercoiled or relaxed DNA substrates at 25 mM NaCl. In lanes 3–8 for each Mlh1–Pms1 was included at 25, 50, 100, 200, 300, or 400 nM. When present ATP or ATPγS was 0.5 mM. Binding reactions were incubated at room temperature for 20 min. Experiments were conducted in triplicate, and representative gels are presented for each condition, illustrating consistent observations across all replicates. (**G**) Modified topology-dependent binding assay reported by Litwin, *et al.* Creation of topoisomer pool and assay conditions are described in the ‘Materials and methods’ section. We were able to resolve seven distinct topoisomers and assigned them arbitrary numbers 0–6 from low to high supercoiling based on relative migration in an agarose gel. The input, supernatants from each separation step, and material where denaturation was incomplete were analyzed by agarose gel. Lane numbers corresponding to analysis in panels (B) and (C) are given. (**H–I**) Representative agarose gel analysis of reaction material. Where included, the concentration of ATP or ATPγS was 0.5 mM. Two hundred nanomolar Mlh1–Pms1 was added in all reactions. (**J**) The amount of each topoisomer was calculated in the bound and unbound fractions and compared to the input. See Supplementary Fig. S3 for quantification details. Because a portion of the bound population resisted proteinase K treatment and heat denaturation, the apparent amount of bound DNA expressed in the plots is input minus the sum of the unbound population for each topoisomer. (**K**) ATP hydrolysis assay measuring pmol of ATP hydrolyzed in a 45 min time period. Reactions contained 400 nM Mlh1–Pms1 and 3.8 nM 4.3 kb pBR322 plasmid either supercoiled or relaxed was included where indicated. The mean and standard deviation for three replicates is reported. (**L**) ATP hydrolysis assays conducted by pre-binding 400 nM Mlh1–Pms1 to 3.8 nM supercoiled or relaxed pBR322 for 15 min, followed by the addition of ATP. Reactions were incubated at 37°C for 45 min. The mean and standard deviation of three replicates is reported. For all panels (J)–(L), showing quantifications, bar graphs represent means with error bars representing standard deviations between experiments. Statistical significance was determined using unpaired *t*-tests with Welch’s correction (Prism 10). Significance is denoted above bars (*P <.05; **P <.005; ***P <.0005; ****P <.0001; ns, not significant).

In the absence of nucleotide, increasing concentrations of Mlh1–Pms1 caused both supercoiled and relaxed plasmids to shift to slower migrating species, consistent with protein binding (Fig. 3A and D). At high Mlh1–Pms1 concentrations, a significant portion of the material shifted into the wells of the gel, likely due to Mlh1–Pms1 forming multimers on the DNA [30–36, 55]. Notably, more material shifted into the wells for supercoiled DNA than for relaxed plasmids, suggesting that supercoiled DNA is likely bound with higher affinity in the absence of ATP. The presence of ATP appeared to have a destabilizing effect on Mlh1–Pms1 binding to supercoiled DNA, whereas it had a minimal effect on binding to relaxed plasmids (Fig. 3B and E). In contrast, the nonhydrolyzable ATP analog ATPγS reduced binding to both DNA substrates (Fig. 3C and F). These results suggest that DNA topology and nucleotide state both influence Mlh1–Pms1 binding.

To further investigate the relationship between DNA topology and nucleotide interactions on Mlh1–Pms1 binding to DNA, we employed a modified high-throughput assay [56] to evaluate its binding to topoisomers of the same plasmid differing only in their writhe number, or degree of supercoiling (Fig. 3G). We prepared a pool of DNA substrates by treating a pUC19 plasmid, where over 90% of the molecules were in a negatively supercoiled state, with a small amount of *E. coli* Topo I (a type I topoisomerase) for 60 s at 25°C followed by heat inactivation. Using this method, we generated seven distinct topoisomers, which were resolved by agarose gel electrophoresis (Fig. 3H, lane 2). These topoisomers were assigned numerical identities from zero (most relaxed) to six (most supercoiled).

We incubated this pool of topoisomers with a FLAG-tagged variant of Mlh1–Pms1 (Mlh1-FLAG-Pms1), under conditions that do not promote endonuclease activity (i.e., no PCNA or manganese were added). Previous studies have shown that this tag does not disrupt Mlh1–Pms1 function [23]. After allowing Mlh1-FLAG-Pms1 to bind to the DNA pool, we used anti-FLAG M2 agarose beads to pull down Mlh1-FLAG-Pms1 and any associated DNA. Following several wash steps, we treated the bead-bound material with proteinase K and then boiled it to release the Mlh1-FLAG-Pms1-bound DNA (Fig. 3G). We ran all material in an agarose gel and quantified the total amount of DNA in each topoisomer band in the unbound and bound fractions after the washing steps relative to the input material to quantify the amount of each topoisomer bound by Mlh1-FLAG-Pms1 (Fig. 3H and I, and Supplementary Fig. S3 for details on quantifications).

Using this method, we found that in the absence of nucleotide, the apparent fraction of DNA bound increased with higher degrees of supercoiling (Fig. 3J, gray bars). When ATP was included in the reaction, there was minimal change in the proportion of relaxed DNA bound by Mlh1–Pms1. However, the apparent proportion of supercoiled DNA bound by Mlh1–Pms1 decreased significantly (Fig. 3J, compare gray bars to maroon bars for each topoisomer). This trend was observed as a function of the degree of supercoiling. Although statistically significant, when ATPγS was included, no apparent difference in binding was observed for Mlh1–Pms1 across DNA topologies, and the amount of DNA pulled down was comparable to that for relaxed plasmid DNA in the absence of nucleotide (Fig. 3J, blue bars). Our data suggest that the protein’s interactions with relaxed DNA are less affected by ATP, indicating that when bound to relaxed DNA, Mlh1–Pms1 may not be in a conformation to bind or hydrolyze ATP efficiently. In contrast, DNA supercoiling creates a sensitivity to ATP that either causes Mlh1–Pms1 to dissociate from the DNA or alters its conformation such that it cannot be effectively pulled down in this assay.

To explore whether the conformation of DNA influences Mlh1–Pms1’s ATPase activity, we performed an ATPase assay using supercoiled and relaxed plasmid substrates. Previous studies have shown that Mlh1–Pms1’s ATPase activity is stimulated by DNA [12, 17, 20], though these experiments used oligonucleotide duplexes. To specifically assess the impact of DNA topology on ATPase activity, we measured ATP hydrolysis using plasmid-based substrates and compared the results to a control reaction containing Mlh1–Pms1 alone in the absence of DNA.

We conducted two variations of this experiment. In the first, ATPase activity was measured when Mlh1–Pms1 was incubated with DNA and ATP simultaneously (Fig. 3K). In the second, Mlh1–Pms1 was pre-incubated with DNA substrates for 15 min before ATP was added, and ATPase activity was measured (Fig. 3L). Larger DNA substrates (4.3 kb pBR322) were used for these experiments, as Mlh1–Pms1 and related ATPases exhibit higher ATPase activity with larger DNA molecules [57]. As expected, some ATP hydrolysis activity was observed in the absence of DNA, consistent with previous studies (Fig. 3K and L, lane 1) [12, 17, 20]. When Mlh1–Pms1, DNA, and ATP were added synchronously, Mlh1–Pms1’s ATPase activity was stimulated to a similar extent by both supercoiled and relaxed plasmids (Fig. 3K). However, when Mlh1–Pms1 was pre-incubated with DNA before ATP addition, supercoiled DNA stimulated ATPase activity to a greater extent than relaxed DNA, reinforcing that Mlh1–Pms1 uses ATP differently on relaxed versus supercoiled DNA (Fig. 3L). These results are consistent with our observations from the topoisomer pull-down assay and the UV crosslinking assay (Fig. 2), where ATP had a larger effect on Mlh1–Pms1’s behavior on supercoiled plasmid compared to relaxed DNA.

### Non-B-form DNA structures can impede endonuclease activation

**Figure 4.**
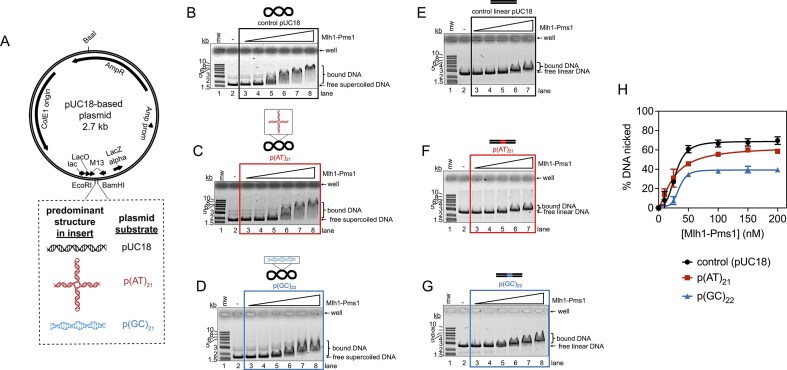
Non-B-form regions disrupt Mlh1–Pms1 activities. (**A**) Dinucleotide repeats were inserted into a 2.7 kb pUC18 plasmid to assess impact on Mlh1–Pms1 activities. Oligonucleotides used to construct the inserts are given in Supplementary Table S1. (**B–D**) Electrophoretic mobility shift assays on supercoiled DNA substrates containing dinucleotide repeat regions. Where indicated, Mlh1–Pms1 concentrations are 25, 50, 100, 200, 300, and 400 nM. Binding reactions were incubated at room temperature for 10 min. Experiments are performed in triplicate and representative gels are included. (**E–G**) DNA binding to linear substrates containing a dinucleotide repeat sequence linearized by BsaI-Hfv2, which positions the dinucleotide repeat sequence in the center of the plasmid. For panels (E) and (F) Mlh1–Pms1 was included at 50, 100, 200, 300, and 400 nM. For panel (G), Mlh1–Pms1 was included at 25, 50, 100, 200, 300, and 400 nM. Experiments are performed in triplicate and representative gels are included. (**H**) Endonuclease assays on substrates containing either no repeat sequence, or a (AT)_21_ or (GC)_21_ repeat sequence. Mlh1–Pms1 was titrated at 10, 25, 50, 100, 150, and 200 nM. The average proportion of supercoiled DNA converted to nicked circular product from three replicates. Error bars are the standard deviation between replicates. Data were fit to a sigmoidal function describing cooperative activity. Representative images are in Supplementary Fig. S5.

Our data suggest that Mlh1–Pms1 may alter DNA conformation. To explore this further, we focused on dinucleotide repeats, which are known to form alternative DNA structures that can significantly affect local DNA topology. We generated circular plasmid-based substrates containing dinucleotide repeat segments to assess Mlh1–Pms1’s ability to induce and respond to these DNA conformational changes (Fig. 4A). Specifically, we introduced a segment of repeating AT units (p(AT)_21_) or GC units (p(GC)_22_) predicted to form cruciform or Z-form structures [58–62]. These substrates were generated by ligating duplex oligonucleotides (Supplementary Table S1) into EcoRI/BamHI-digested pUC18.

To verify approximately how much of each plasmid contains a cruciform structure, we used the structure-selective T7 endonuclease I, which recognizes Holliday junctions and cruciform structures. Control plasmids without dinucleotide repeat inserts were not cleaved by T7 endonuclease I in either their supercoiled or linear forms (Supplementary Fig. S4A and D). In contrast, the supercoiled p(AT)_21_ plasmid was efficiently cleaved by T7 endonuclease I (nearly 80% of supercoiled molecules), resulting in linearization, consistent with the formation of a cruciform structure (Supplementary Fig. S4B and D). When the plasmid was linearized, placing the dinucleotide repeat centrally within the linear plasmid, only ∼10% of the molecules were cleaved by T7 endonuclease I (Supplementary Fig. S4B and D). These findings indicate that the cruciform structure is found in a majority of molecules in the supercoiled form but is present in only a fraction of the molecules when the plasmid is linearized. For the p(GC)_22_ plasmid, ∼25% of the supercoiled molecules were cleaved by T7 endonuclease I, resulting in linearization. However, no detectable cleavage was observed in the linearized p(GC)_22_ plasmid (Supplementary Fig. S4C and D). This suggests that the supercoiled p(GC)_22_ plasmid may adopt a mixture of conformations, including a Z-form structure and a cruciform [61], but the cruciform structure is minimally present.

Using an electrophoretic mobility shift assay, we measured Mlh1–Pms1’s affinity for the supercoiled forms of these substrates. We found that Mlh1–Pms1 bound the p(AT)_21_ plasmid similarly to the control pUC18 plasmid (Fig. 4B and C). In contrast, the p(GC)_22_ plasmid displayed a less prominent gel shift compared to the other substrates, suggesting that the p(GC)_22_ plasmid is either a lower-affinity substrate or destabilizes the Mlh1–Pms1 multimer relative to plasmids without this insert (Fig. 4D). This weaker binding state appears to be dependent on the insert being in a topologically closed system, as we observed little difference in apparent affinities among the substrates when they were tested in their linear forms (Fig. 4E–G).

A possible explanation for the weaker binding observed with the supercoiled p(GC)_22_ substrate is that this segment is predicted to form a left-handed Z-form structure. When embedded into a plasmid that is otherwise predominantly a right-handed B-form helix, this structural feature likely alters the overall biophysical parameters (e.g. twist, writhe, linking number) of the plasmid relative to the control pUC18 plasmid. Additionally, a region near the insert may become significantly deformed to maintain supercoiling in the remainder of the plasmid. This alteration in topology and conformation may result in a substrate that is less favorable for Mlh1–Pms1 binding.

We also measured endonuclease activity on the supercoiled versions of these substrates. While we observed no significant difference in the apparent affinity of Mlh1–Pms1 for the p(AT)_21_ plasmid compared to the pUC18 control plasmid, there was a reduction in the proportion of DNA nicked by Mlh1–Pms1 for the p(AT)_21_ plasmid (Fig. 4H and Supplementary Fig. S5). The p(GC)_22_ plasmid exhibited an even greater reduction in nicking activity compared to both the pUC18 and p(AT)_21_ plasmids. Together, these data suggest that structures formed by dinucleotide repeats, such as cruciform and Z-form segments, alter local DNA topology in ways that impact Mlh1–Pms1’s affinity for and dynamics on DNA, which are critical for its function.

### Mlh1 and Pms1 use their ATPase activities to alter DNA structure

To better understand how Mlh1–Pms1 may alter the shape of DNA, use ATP in this process, and how this may influence endonuclease activation, we leveraged our previous observation that Mlh1–Pms1 can stimulate *E. coli* Topo I, a type I topoisomerase [35]. Previously, we hypothesized that this stimulation was due to Mlh1–Pms1 promoting DNA–DNA associations, thereby generating a substrate more favorable for the topoisomerase. Expanding on this, we aimed to determine whether Mlh1–Pms1 could similarly influence the activity of other enzymes that alter DNA topology and to what extent ATPase activity was required for these effects.

**Figure 5.**
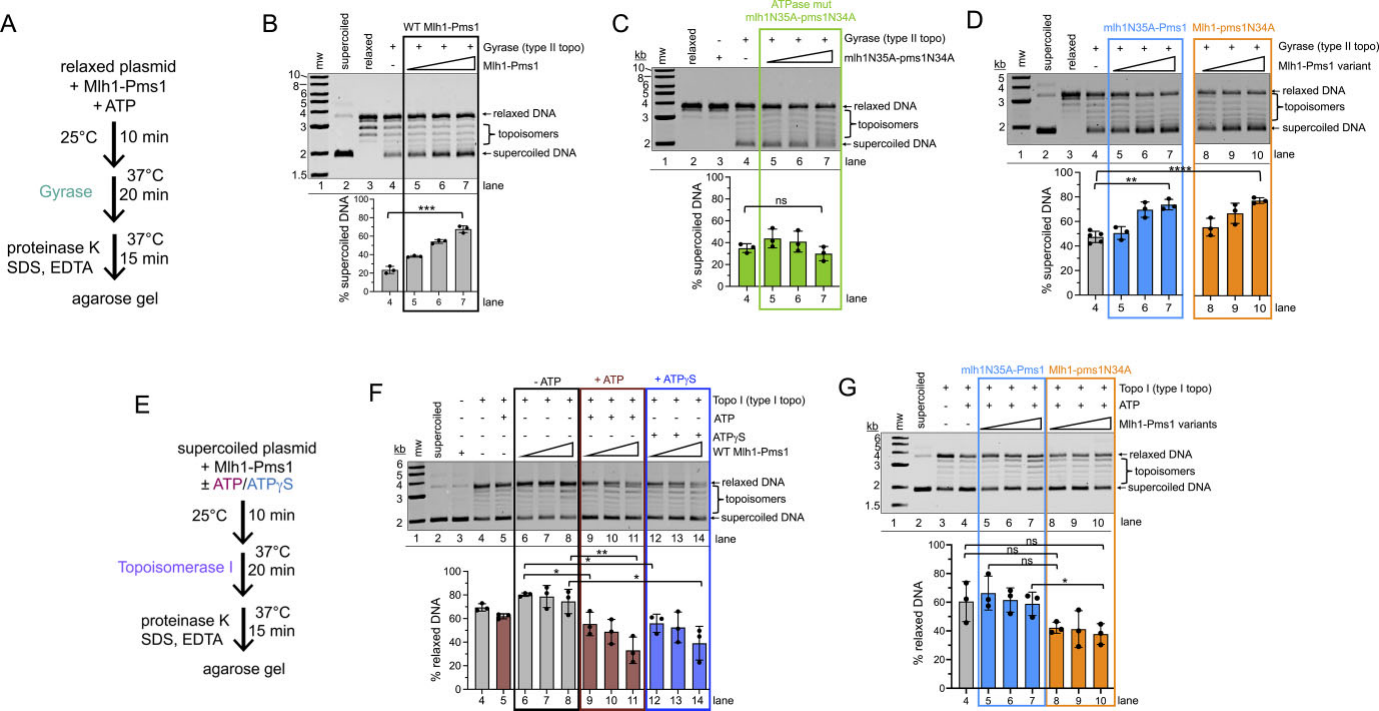
Mlh1–Pms1 enhances Topoisomerase I and Gyrase, with distinct roles for ATP. (**A**) Schematic for assay probing for *E. coli* Gyrase enhancement. Relaxed substrate was prepared by incubating supercoiled DNA with a topoisomerase, then inactivating the topoisomerase, see the ‘Materials and methods’ section for details. The relaxed circular DNA was then incubated with Mlh1–Pms1 or Mlh1–Pms1 ATPase mutants (titrated at 50, 100, or 200 nM) in the presence of 1 mM ATP, followed by the addition of *E. coli* Gyrase. Reaction products were analyzed by agarose gel. (**B–D**) The average proportion of supercoiled product relative to the total amount of DNA in each lane is reported for triplicate experiments. Reactions were performed at 24 mM KCl. Error bars represent the standard deviation between experiments. (**E**) Schematic for assay probing *E. coli* Topo I enhancement. Supercoiled DNA was incubated with Mlh1–Pms1 (titrated at 50, 100, or 200 nM where indicated) in the presence or absence of 0.5 mM ATP or ATPγS, followed by the addition of Topo I from *E. coli*. Reaction products were analyzed by agarose gel. (**F**) Summary data from agarose gel analysis of assay described in panel (E) using wild-type Mlh1–Pms1. (**G**) Summary of agarose gel analysis of assay described in panel (E) using mlh1N35A-Pms1 or Mlh1-pms1N34A ATPase mutants. For panels (F) and (G), the average proportion of relaxed circular DNA product relative to the total amount of DNA in each lane is reported for triplicate experiments. Reactions were performed at 50 mM potassium acetate. Error bars represent the standard deviation between experiments. For quantified data, bar graphs represent means with error bars representing standard deviations between experiments. Statistical significance was determined using unpaired *t*-tests with Welch’s correction (Prism 10). Significance is denoted above bars (*P <.05; **P <.005; ***P <.0005; ****P <.0001; ns, not significant).

Consistent with its stimulatory effect on *E. coli* Topo I, Mlh1–Pms1 was also able to enhance the activity of *E. coli* Gyrase, a type II topoisomerase that converts relaxed plasmids to supercoiled topologies (Fig. 5A and B). Since Gyrase uses ATP hydrolysis to convert relaxed DNA into supercoiled DNA, we next tested whether ATP hydrolysis by Mlh1–Pms1 was necessary for this stimulation. For this, we used an Mlh1–Pms1 mutant (mlh1N35A-pms1N34A) that cannot bind or hydrolyze ATP in either subunit [17, 18, 24]. In the presence of this mutant, we no longer observed enhancement of DNA supercoiling compared to the negative control, suggesting that Mlh1–Pms1’s ability to stimulate Gyrase activity requires ATP binding and subsequent rearrangement of the DNA (Fig. 5C). When we performed this assay with mutants where ATP binding in individual subunits was inhibited (mlh1N35A-Pms1 and Mlh1-pms1N34A), we found that when ATP binding in either subunit was eliminated, Gyrase could still be stimulated to supercoil the DNA (Fig. 5D, compare lanes 5–7 and 8–10 with lane 4). These data suggest that Mlh1–Pms1 may need its ATPase to manipulate DNA, but that ATPase activity in either Mlh1 or Pms1 is sufficient for this process.

To also determine the roles of individual subunits and the effects of ATP on Mlh1–Pms1’s ability to stimulate *E. coli* Topo I, we performed the previously reported topoisomerase assay with the modification that we used 50-fold less topoisomerase to keep the degree of stimulation within the linear range, amplifying subtle effects (Fig. 5E and F) [35]. When either ATP or ATPγS was added to this assay under these conditions, we observed a decrease in the formation of the relaxed circular DNA product compared to equivalent protein concentrations without nucleotide (Fig. 5F, compare lanes 9–11 and 12–14 with 6–8). These data suggest that Mlh1–Pms1 in the ATP-bound state inhibits topoisomerase activity.

To investigate the role of each ATPase site in the enhancement of Topo I, we used the Mlh1–Pms1 mutants where ATP binding was abolished in each subunit individually. ATP was included in all reactions. In the absence of ATP binding by the Mlh1 subunit (mlh1N35A-Pms1), we observed no significant inhibition of topoisomerase or stimulation compared to the control (Fig. 5G, compare lanes 5–7 with 4). When Mlh1–Pms1 containing an ATP-binding mutation in the Pms1 subunit was used (Mlh1-pms1N34A), we observed a slight, but insignificant decrease in the amount of DNA converted to relaxed product compared to reactions without Mlh1–Pms1 (Fig. 5G, compare lanes 8–10 with 4 and 5–7), suggesting that ATPase activity in both subunits may be contributing to the ATP-dependent inhibition observed in Fig. 5F.

### Mlh1–Pms1 reshapes DNA locally at nicks and supercoils

**Figure 6.**
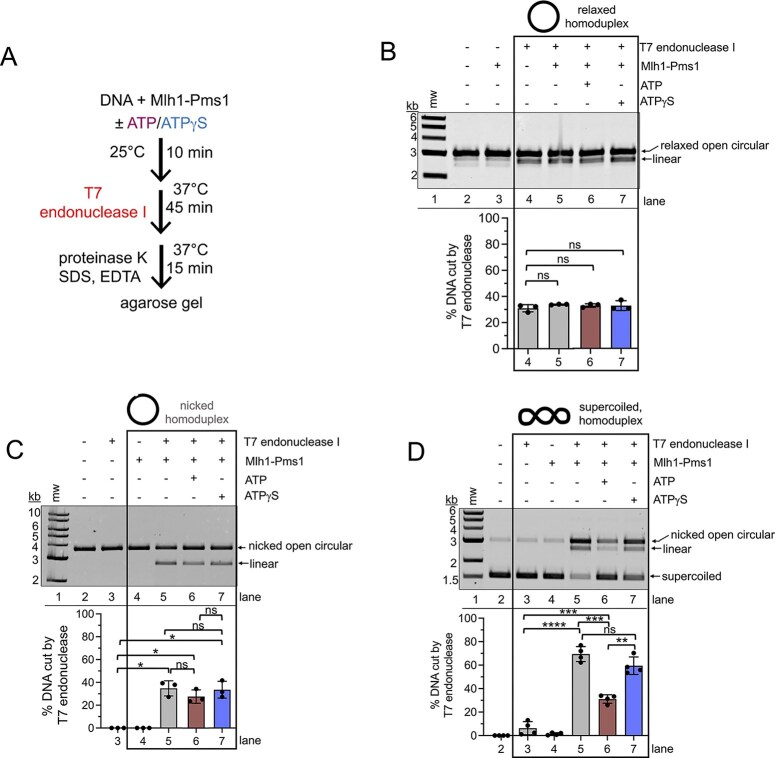
Mlh1–Pms1 can rearrange DNA into a structure recognizable by a structure-selective endonuclease. (**A**) Schematic for structure-selective T7 endonuclease I stimulation assay (see the ‘Materials and methods’ section for additional details). (**B**) Reactions with relaxed plasmid DNA (generated by topoisomerase) contained 100 nM Mlh1–Pms1, 0.5 mM ATP or ATPγS, and 0.6 units of T7 endonuclease I where indicated. The amount of DNA linearized was determined from three independent experiments. (**C**) Reactions with nicked plasmid DNA (generated with Nt.BspQI) contained 100 nM Mlh1–Pms1, 0.5 mM ATP or ATPγS, and 0.6 units of T7 endonuclease I where indicated. All reactions contained 50 mM NaCl. For each condition, the fraction of nicked DNA converted to linear product was quantified across three independent experiments. (**D**) Reactions with supercoiled plasmid DNA contained 50 nM Mlh1–Pms1, 0.5 mM ATP or ATPγS, and 0.2 units of T7 endonuclease I where indicated. The fraction of supercoiled DNA converted to nicked open circular or linear product was quantified across four independent experiments. For all panels, bar graphs show mean values with error bars representing standard deviations. Statistical significance was determined using unpaired *t*-tests with Welch’s correction (Prism 10). Significance is denoted above bars (*P <.05; **P <.005; ***P <.0005; ****P <.0001; ns, not significant).

Our assays in Figs 2, 5, and 6, which probe DNA conformational changes induced by Mlh1–Pms1, support a role for ATP in remodeling DNA topology on a global scale. Because nicks appear to block these global compaction events, we next asked whether Mlh1–Pms1 also promotes local distortions in DNA, particularly at nicks where global remodeling is suppressed. To address this, we examined stimulation of T7 endonuclease I, a nuclease that cleaves branched or cruciform-like structures and nicked DNA (Fig. 6A).

On relaxed continuous DNA, Mlh1–Pms1 did not stimulate T7 endonuclease I cleavage under any nucleotide condition (Fig. 6B). Although it should be noted that our assay could not detect additional single-strand nicks that might occur from T7 endonuclease activity, it could measure double-strand breaks by observing the conversion of the open circular form to a linear form. When we used this same plasmid, but containing a pre-existing nick at a single site, we found that Mlh1–Pms1 stimulated the structure-selective activity of T7 endonuclease I, resulting in an increased linear DNA product (Fig. 6C, compare lanes 3 and 5). Including ATP or ATPγS had minimal effects on the stimulation (Fig. 6C, lanes 6 and 7), suggesting that once global compaction is inhibited by binding a nick, Mlh1–Pms1 may promote local DNA distortions in an ATP-independent manner.

We next incubated supercoiled DNA with Mlh1–Pms1, followed by the addition of T7 endonuclease I to assess DNA cleavage. Without Mlh1–Pms1, T7 endonuclease minimally cut the DNA (Fig. 6D, compare lanes 2 and 3), consistent with the occasional formation of looped or cruciform-like structures due to supercoiling. Under these conditions, it is clear that Mlh1–Pms1 itself is not cleaving DNA because Mn^2+^ and RFC/PCNA, which are required for its endonuclease activity, were omitted (Fig. 6D, lane 4) [10, 20, 48, 73]. When Mlh1–Pms1 was included prior to adding T7 endonuclease I, a significant increase in DNA cleavage was observed, suggesting that Mlh1–Pms1 may rearrange DNA into structures that are substrates for T7 endonuclease I (Fig. 6D, lane 5). Unlike the global compaction observed in UV-crosslinking assays, this activity did not require ATP–ATP slightly reduced stimulation (Fig. 6D, lane 6), and ATPγS did not alter cleavage compared to nucleotide-free reactions (Fig. 6D, lane 7). These results suggest that on supercoiled DNA, Mlh1–Pms1 may promote local junction-like distortions without an ATP requirement, consistent with our earlier observation that ATP binding and hydrolysis drive dissociation from supercoiled DNA (Fig. 3).

### Mlh1–Pms1 protects nick sites from RFC

**Figure 7.**
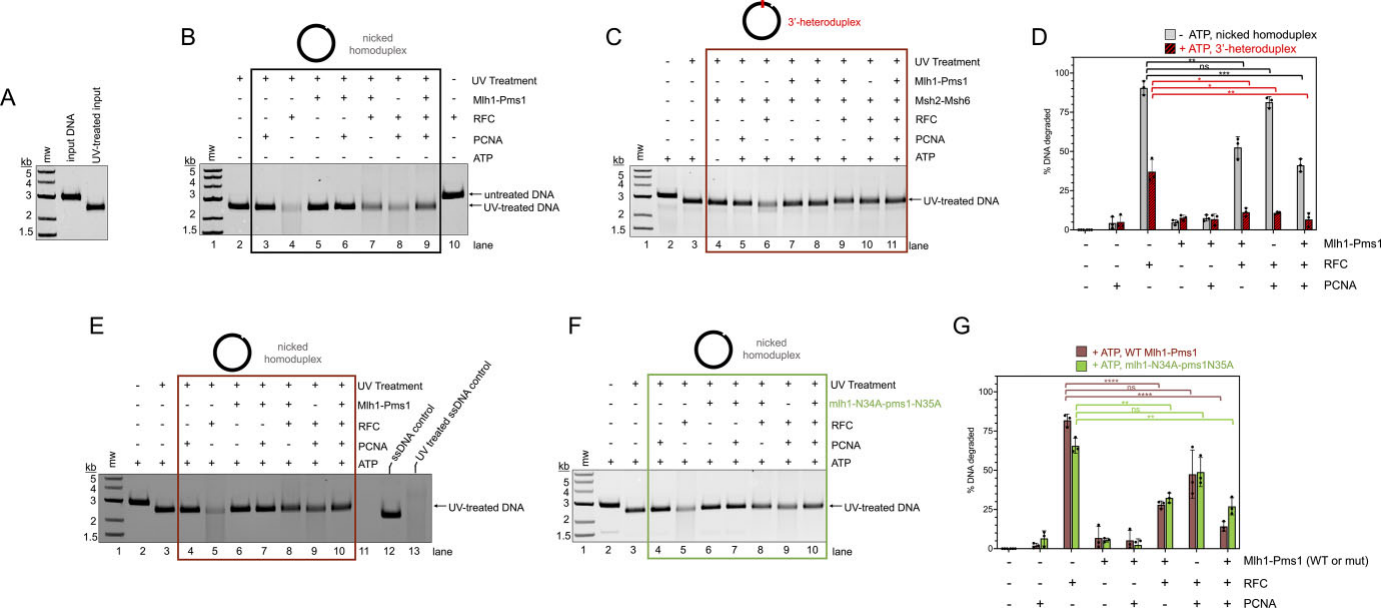
Mlh1–Pms1 and RFC compete on nicked DNA substrates. (**A**) UV treatment of nicked DNA substrates results in linearization. (**B, C, E, F**) A 2.7-kb nicked plasmid, either homoduplex or heteroduplex (as indicated), was incubated with wild-type Mlh1–Pms1 or the mlh1-N34A-pms1-N35A mutant (100 nM), RFC (100 nM), PCNA (500 nM), and, where applicable, Msh2–Msh6 (50 nM), in the presence or absence of 0.5 mM ATP. No MnSO_4_ was included in any reaction. All reactions included 150 mM NaCl. Samples were then UV-irradiated, deproteinated, and analyzed by agarose gel electrophoresis as in Fig. 2A. (**D, G**) Quantification of DNA degradation, measured as loss of signal in the UV-treated DNA band relative to negative controls. Data represent mean values ± standard deviation from three independent experiments. Statistical significance was determined by unpaired *t*-tests with Welch’s correction in Prism 10 and is denoted above bars (*P <.05; **P <.005; ***P <.0005; ****P <.0001; ns, not significant).

Using the UV-based structural probing assay described in Fig. 2A, we next asked whether Mlh1–Pms1 plays a functional role at DNA nicks and how local distortions at these sites may influence other mismatch repair proteins. We observed that irradiation of plasmid DNA containing a pre-existing nick converted much of the substrate into a form migrating similarly to linear DNA, consistent with a UV-induced break occurring on the strand opposite the nick (Fig. 7A). When RFC was added, with or without PCNA, the plasmid appeared to be further damaged and degraded (Fig. 7B, lanes 4 and 8 relative to 2). This effect likely reflects RFC binding at the nick, which promotes partial strand melting and renders the DNA at this site more susceptible to UV-induced breaks [74–78]. Consistent with this, no degradation was observed in the absence of UV irradiation (Fig. 7B, lane 10), ruling out contaminating nuclease activity. Additionally, MnSO_4_ was omitted from reactions to prevent nonspecific nicking by Mlh1–Pms1 (Supplementary Fig. S2A and B).

Inclusion of Mlh1–Pms1 attenuated this RFC-dependent degradation (Fig. 7B, lanes 7 and 9; quantified in Fig. 7D), suggesting that Mlh1–Pms1 may compete for access to the nick and stabilize it against RFC-promoted melting. This is consistent with prior evidence that Mlh1–Pms1 can bind to nick sites and can block access by other proteins [29, 79]. When the assay was performed on a 3′-heteroduplex substrate under conditions similar to those used in Fig. 1 that localized mismatch repair factors near the mismatch, RFC-induced degradation was reduced but still present, and Mlh1–Pms1 retained the ability to protect the nick (Fig. 7C and D). Addition of ATP modestly reduced the extent of degradation (Fig. 7E and G), likely reflecting increased dynamics of RFC at nick sites rather than a major role for ATP in Mlh1–Pms1 protection, because similar results were obtained with the Mlh1–Pms1 ATP hydrolysis-deficient mutant (Fig. 7F and G). This is also consistent with past work showing that Mlh1–Pms1 does not use its ATPase activity to recognize pre-existing nicks [29]. Together, these findings suggest that Mlh1–Pms1 may stabilize nicked DNA against RFC-promoted melting, most likely by directly competing with RFC for access to DNA nicks.

### Timing of encountering a pre-existing nick affects Mlh1–Pms1’s endonuclease activity

**Figure 8.**
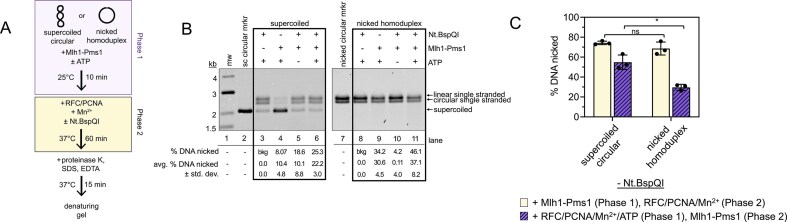
Mlh1–Pms1 preferentially incises DNA with a pre-existing nick when bound before RFC/PCNA. (**A**) Schematic of the phased endonuclease assay used to test how the timing of nick formation influences Mlh1–Pms1 activity. (**B**) Assay in which plasmid DNA was pre-incubated with Mlh1–Pms1 (75 nM) for 10 min before adding RFC (100 nM), PCNA (500 nM), Nt.BspQI (10 units), and 2.5 mM MnSO_4_, to initiate endonuclease activity in a buffer containing 20 mM KCl. ATP (0.5 mM) was included where indicated to test nucleotide effects. % DNA nicked was measured as loss of single stranded bands relative to negative controls in lanes 3 and 8. All lanes are *n* = 3. (**C**) Parallel assays performed as in panels (A) and (B) but with 200 nM Mlh1–Pms1. In one control, Nt.BspQI was omitted (light yellow), resulting in Mlh1–Pms1 prebinding before RFC/PCNA and MnSO_4_ were added. In the second control, both Nt.BspQI and the two-phase design were omitted by adding RFC/PCNA and Mlh1–Pms1 simultaneously (purple, striped bars). Quantification shows mean values from three independent experiments. Statistical significance was determined as in other assays (see Supplementary Fig. S6 for representative gels and Mlh1–Pms1 titration data).

Because Mlh1–Pms1 can compete with RFC for access to DNA nicks, we next asked how the timing of Mlh1–Pms1 encountering a pre-existing nick influences Mlh1–Pms1 endonuclease stimulation by RFC-loaded PCNA. To address this, we designed a phased nicking experiment in which Mlh1–Pms1 was pre-bound to either supercoiled or pre-nicked plasmids before the sequential addition of RFC/PCNA, divalent metal, and, in some cases, a restriction nicking enzyme (Fig. 8A and Supplementary Fig. S6A). This approach allowed us to directly compare outcomes when Mlh1–Pms1 engaged a pre-existing nick prior to RFC/PCNA versus when a nick was introduced only after these factors were already bound.

Using this assay, we observed that on supercoiled DNA, when Mlh1–Pms1 was pre-bound before the addition of RFC/PCNA and the introduction of a discrete nick by Nt.BspQI, the plasmid was nicked with modest efficiency (Fig. 8B, compare lanes 3 and 6). In contrast, when the plasmid was nicked before Mlh1–Pms1 binding, the amount of DNA nicked by Mlh1–Pms1 increased relative to the above supercoiled control, suggesting that direct engagement of a pre-existing nick primes Mlh1–Pms1 for endonuclease activation (Fig. 8B, compare lanes 8 and 11). When DNA was pre-bound by Mlh1–Pms1 prior to RFC/PCNA addition and Nt.BspQI was omitted, the pre-nicked substrate was nicked more efficiently than the supercoiled plasmid (Fig. 8B, compare lanes 4 and 9). This observation contrasts with previous reports where nicked plasmids were nicked less efficiently than continuous DNA [29, 35]. In those studies, either RFC/PCNA and Mlh1–Pms1 were added simultaneously, or RFC was omitted and PCNA was instead loaded by threading onto the ends of linearized plasmids. Taken together, these observations suggest that RFC and Mlh1–Pms1 may compete for access to nicked DNA and that pre-binding of Mlh1–Pms1 to nicks can enhance the overall nicking reaction.

When the assay was performed without introducing a nick in the second phase, both substrates were nicked to approximately the same extent at higher concentrations of Mlh1–Pms1 (Fig. 8C, yellow bars, and Supplementary Fig. S6B). However, when RFC/PCNA, ATP, and Mn^2+^ were pre-bound to nicked DNA prior to the addition of Mlh1–Pms1, the endonuclease activity of Mlh1–Pms1 was suppressed more strongly than when Mlh1–Pms1 was pre-bound to the DNA, relative to supercoiled DNA (Fig. 8D, compare differences in purple to yellow bars, and Supplementary Fig. S6C). These results suggest that the order of protein engagement at a nick dictates whether Mlh1–Pms1 is activated or suppressed, consistent with a model in which productive incision requires Mlh1–Pms1 binding the nick before PCNA loading.

## Discussion

**Figure 9.**
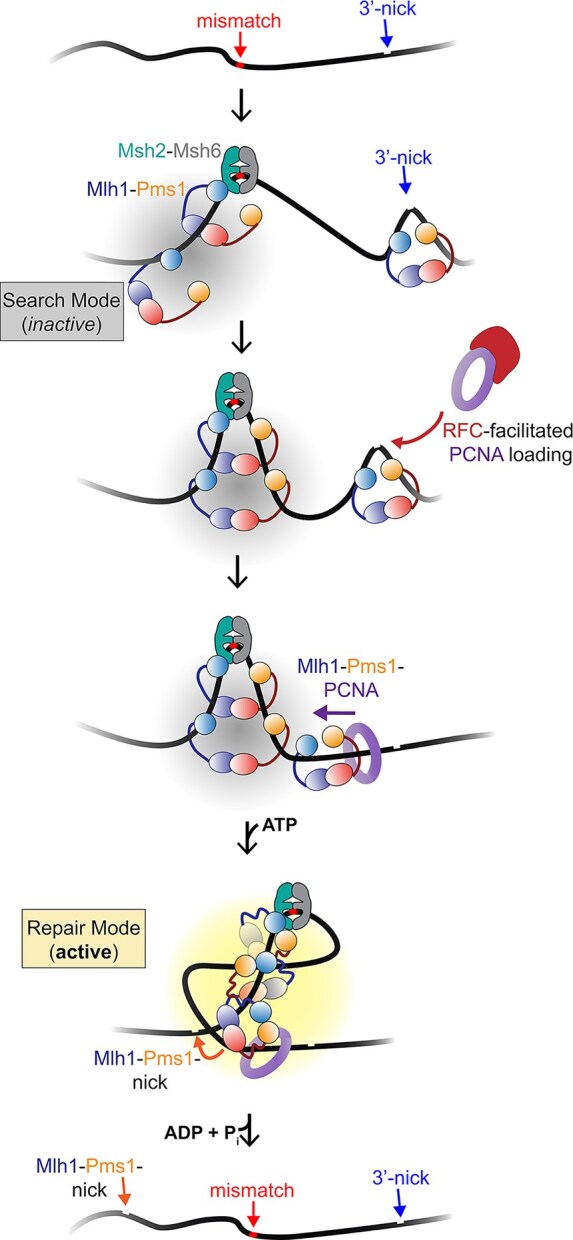
Model for DNA rearrangement in mismatch repair. Mlh1–Pms1 is recruited to mismatched DNA through interactions with Msh2–Msh6 and can compact DNA. When Mlh1–Pms1 recognizes a pre-existing nick, it can locally distort the DNA but does not promote global compaction. Instead, nick-bound Mlh1–Pms1 may facilitate PCNA loading. Once loaded, PCNA-associated Mlh1–Pms1 can interact with Mlh1–Pms1 proteins bound near the mismatch. ATP binding then stimulates a global DNA rearrangement that mimics a supercoiled-like structure, potentially allowing PCNA-bound Mlh1–Pms1 to bridge sites distant from both the mismatch and the pre-existing nick. PCNA interactions also stimulate Mlh1–Pms1 endonuclease and ATPase activities, which may destabilize and dissociate Mlh1–Pms1 from the DNA, leaving the substrate accessible for mismatch removal and subsequent repair.

Our data support a model in which Mlh1–Pms1 is recruited to mismatched DNA by Msh2–Msh6 (Fig. 1B and C). A pre-existing nick enhances Mlh1–Pms1 localization, likely stabilizing the complex near the mismatch and along the intervening DNA contour. We observed ATP-dependent DNA compaction on continuous DNA, but this activity was disrupted by pre-existing nicks (Figs 2 and 5). Similar inhibition of Mlh1–Pms1 function by nicks has been reported previously, where nicks partially reduced endonuclease activity on linear DNA with self-loading PCNA and suppressed ATPase activity on plasmid substrates with or without pre-loaded PCNA, although nicked DNA still served as a suitable binding substrate and appeared to act as a recognition site [29, 35]. Our data suggest that Mlh1–Pms1 recognizes nicks, distorts them locally, and protects them from other factors (Figs 6–8), but may ultimately use these sites to aid PCNA loading (Fig. 9).

These findings may inform models for strand discrimination in mismatch repair. PCNA has been shown to act as a strand-discrimination signal that biases which DNA strand Mlh1–Pms1 incises and which strand is subsequently repaired [10, 11, 13, 14, 48, 73, 80, 81]. However it remains unclear whether the repair-associated PCNA is inherited directly from the replisome or loaded *de novo* by RFC in the vicinity of repair sites [36, 81]. Our data suggest that Mlh1–Pms1’s engagement with nicks could facilitate either pathway, establishing a site that ensures PCNA is positioned to direct strand-specific incision regardless of its origin.

This model is further supported by experiments showing that Mlh1–Pms1 protects nicked DNA from RFC, yet nicked DNA bound by Mlh1–Pms1 prior to RFC/PCNA addition was nicked more efficiently than DNA nicked simultaneously with RFC/PCNA (Fig. 8B). Altering the order of addition revealed that when RFC/PCNA were introduced before Mlh1–Pms1, endonuclease activity on nicked DNA was suppressed more strongly than on supercoiled DNA, suggesting direct competition between Mlh1–Pms1 and RFC for nick access. In contrast, when Mlh1–Pms1 encountered the nick first, endonuclease activity was significantly higher. Because nicking only occurred when PCNA was included, these results point to a possible handoff of PCNA from RFC to Mlh1–Pms1 at pre-existing nick sites, thereby stimulating endonuclease activity when Mlh1–Pms1 arrives before RFC. Alternatively, if RFC loads PCNA prior to Mlh1–Pms1 binding (Fig. 8C), Mlh1–Pms1 associates with the nick site but cannot be further stimulated by PCNA, consistent with previous observations that nicks can inhibit endonuclease activity. Finally, the idea that Mlh1–Pms1 creates a PCNA loading site for RFC is supported by our finding that nick-bound Mlh1–Pms1 induces local DNA distortions detectable by stimulation of T7 endonuclease I (Fig. 6C).

We propose that mismatch-proximal Mlh1–Pms1 complexes may compact DNA in an ATP-dependent search for the PCNA-licensed Mlh1–Pms1 complex (Fig. 9). Successive rounds of Msh2–Msh6–mediated recruitment coupled to ATP binding and hydrolysis may drive this search process, and such compaction could transiently trap Msh2–Msh6 at the mismatch site, restraining it until downstream incision occurs [33, 82]. Our model is supported by our observations that Mlh1–Pms1 may generate DNA resembling supercoiled structures, which stimulate ATPase activity and protein dissociation (Figs 3 and 5). We further found that pre-existing nicks suppress DNA compaction but enhance local distortions measured by T7 endonuclease I stimulation (Fig. 6C), consistent with a model in which mismatch-bound complexes generate global rearrangements, while nick-bound complexes induce local remodeling. Once a mismatch-associated complex encounters a nick-associated complex, ATP binding may induce DNA rearrangements that align the duplex for incision 5′ to the mismatch.

These findings also emphasize the importance of DNA length and topology. Compaction and rearrangement appear to require DNA substrates exceeding the persistence length, explaining why plasmids or long linear DNA are needed for robust *in vitro* endonuclease activity [34, 35]. Perturbations that alter DNA topology, such as Z-form or cruciform inserts, impaired Mlh1–Pms1 remodeling and nicking (Fig. 4), suggesting that alternative structures interfere with ATP-driven rearrangements required for endonuclease activity. This sensitivity to DNA architecture may contribute to the elevated instability of microsatellite regions, which frequently adopt non-B DNA conformations. Our findings therefore extend the classical view that microsatellite instability arises solely from polymerase errors by suggesting that mismatch repair itself may be less efficient in these regions due to compromised Mlh1–Pms1 function.

ATP plays multiple roles in our model. Endonuclease activity *per se* does not require ATP hydrolysis [20, 29], but ATP binding is essential for compaction and rearrangement, while hydrolysis destabilizes protein–DNA interactions and promotes release. Our binding and ATPase assays support this division of labor: ATP reduced pull-down on supercoiled substrates, supercoils stimulated ATP turnover, and ATP enhanced dissociation (Fig. 3A–F and H–L). These data suggest a “catch-and-release” cycle in which ATP binding drives compaction and hydrolysis recycles the complex. The dissociative effects of ATP in these reactions potentially align with sliding clamp models for MutL, in which disassembly of a searching complex may involve nucleotide-bound proteins sliding away from the site of mismatch localization [8, 37, 83]. Subsequent ATP hydrolysis could then promote full dissociation from DNA [40]. Interactions with PCNA further enhance ATPase activity [12], consistent with a role for PCNA in stimulating release following incision. The intrinsically disordered regions of Mlh1–Pms1 likely mediate these conformational transitions, as suggested by earlier work [17–23]. Without ATP-driven contortions, Mlh1–Pms1 may become trapped on DNA, consistent with the mutator phenotypes of ATP-binding mutants [24, 25] and protection assays showing impaired recycling [29].

Finally, our data may reflect conserved features across MutL homologs. Atomic force microscopy studies of human MLH1–PMS2 reveal DNA compaction footprints of 50–300 base pairs when ATP is present, consistent with our observations and with the distances between mismatches and incisions measured *in vivo* [10, 11, 33]. Yeast Mlh1–Mlh3 shows similar ATP-mediated conformational changes and cooperative DNA interactions [84], suggesting that DNA reshaping is a general function of MutL proteins adapted to different contexts, from mismatch repair to meiotic recombination and trinucleotide repeat instability.

In summary, our results suggest a three-state cycle in which Mlh1–Pms1 transitions from an ATP-driven DNA compaction state near mismatches (search), to a nick-bound pause that stabilizes strand discrimination signals, and finally to a PCNA-licensed activation state that promotes incision. This framework reconciles why ATP hydrolysis is essential *in vivo* but dispensable for nicking *in vitro*, highlights how DNA topology and alternative structures regulate repair efficiency, and provides a mechanistic basis for the integration of mismatch recognition, strand discrimination, and endonuclease activation in eukaryotic mismatch repair.

## Supplementary Material

gkaf1252_Supplemental_File

## Data Availability

The data underlying this article are available in the article and in its supplementary material.
