# Supplementary material for: Mlh1–Pms1 couples ATP-driven DNA compaction with nick-dependent endonuclease activation
Source: Nucleic Acids Res. 2025 Dec 3;53(22):gkaf1252. doi: 10.1093/nar/gkaf1252 (PMC12673852; doi:10.1093/nar/gkaf1252)
Supplement: gkaf1252_Supplemental_File [file gkaf1252_supplemental_file.pdf]

SUPPLEMENTARY MATERIAL

Mlh1-Pms1 Couples ATP-Driven DNA Compaction with Nick-Dependent Endonuclease Activation

AUTHORS

Bryce W. Collingwood[1], Amruta N. Bhalkar[1], Carol M. Manhart[1, *]

[1]Department of Chemistry, Temple University, Philadelphia, Pennsylvania, 19122, USA
*To whom correspondence should be addressed. Tel: 215-204-7167; Email: carol.manhart@temple.edu

SUPPLEMENTARY TABLES

Oligonucleotide Number	Substrate	Oligonucleotide sequence (5' to 3')
CMO336	p(AT)$_{21}$	GATCCGCTCTTCGATCATATATATATATATATATATATAT ATATATATATATATATATGCTCTTCAGG
CMO337	p(AT)$_{21}$	AATTCCTGAAGAGCTATATATATATATATATATATATATA TATATATATATATATAGATCGAAGAGCG
CMO398	p(GC)$_{22}$	GATCCGCTCTTCGATCGCGCGCGCGCGCGCGCGCGCGCGC GCGCGCGCGCGCGCGCGCGCTCTTCAGG
CMO399	p(GC)$_{22}$	AATTCCTGAAGAGCCGCGCGCGCGCGCGCGCGCGCGCGCG CGCGCGCGCGCGCGCGGATCGAAGAGCG
CMO487	Heteroduplex	[P]-CGGTACCCGGGG<u>G</u>TCCTCTAGAGTC

Table S1. Oligonucleotides used to construct plasmid substrates containing non-B-form segments and heteroduplex plasmid substrates. For CMO487, [P] indicates a 5'-phosphate and the position of the mismatch is indicated in red, underlined text . All oligonucleotides were obtained commercially from Integrated DNA Technologies. See Materials and Methods for details on substrate construction.

substrate	RE	Mlh1-Pms1	Msh2-Msh6	p-value
3’-nicked heteroduplex	HindIII	-	-	< 0.0001
		+	+	
		+	-	< 0.0001
		+	+	
		-	+	0.0141
		+	+	
nicked homoduplex	HindIII	-	-	0.0059
		+	+	
		+	-	0.0055
		+	+	
		-	+	0.0829
		+	+	
continuous heteroduplex	HindIII	-	-	0.0050
		+	+	
		+	-	0.0150
		+	+	
		-	+	0.3755
		+	+	
		-	-	0.0027
		-	+	
3’-nicked heteroduplex	EcoRI	-	-	0.0268
		+	+	
		+	-	0.0365
		+	+	
		-	+	0.0163
		+	+	
nicked homoduplex	EcoRI	-	-	0.0205
		+	+	
		+	-	0.0162
		+	+	
		-	+	0.2518
		+	+	
		-	-	0.0701
		-	+	
		-	+	0.2518
		+	+	
continuous heteroduplex	EcoRI	-	-	0.0063
		+	+	
		+	-	0.0163
		+	+	
		-	+	0.9096

		+	+	
		-	-	0.0101
		-	+	
		-	+	0.9096
		+	+	
3'-nicked heteroduplex	HindIII<br>EcoRI	+	+	0.0119
nicked homoduplex	HindIII<br>EcoRI	+	+	0.0331
continuous heteroduplex	HindIII<br>EcoRI	+	+	0.0086
3'-nicked heteroduplex<br>nicked homoduplex	HindIII	+	+	0.0006
3'-nicked heteroduplex<br>continuous heteroduplex	HindIII	+	+	0.0008
nicked homoduplex<br>continuous heteroduplex	HindIII	+	+	0.0058
3'-nicked heteroduplex<br>nicked homoduplex	EcoRI	+	+	0.5051
3'-nicked heteroduplex<br>continuous heteroduplex	EcoRI	+	+	0.1325
nicked homoduplex<br>continuous heteroduplex	EcoRI	+	+	0.0697
3'-nicked heteroduplex	XmnI	-	-	0.1851
		+	-	
		-	-	0.0290
		-	+	
		-	-	0.1234
		+	+	
		-	+	0.6041
		+	+	
	XmnI	-	-	0.2539

nicked homoduplex		+	-	
		-	-	0.0358
		-	+	
		-	-	0.0007
		+	+	
		-	+	0.1764
		+	+	
3'-nicked heteroduplex	XmnI	+	+	0.2477
nicked homoduplex				

Table S2. Statistical differences between conditions for restriction enzyme protection assays in Figure 1 and Figure S1. Statistical analysis was performed using unpaired t-tests with Welch's correction (Prism 10).

SUPPLEMENTARY FIGURES

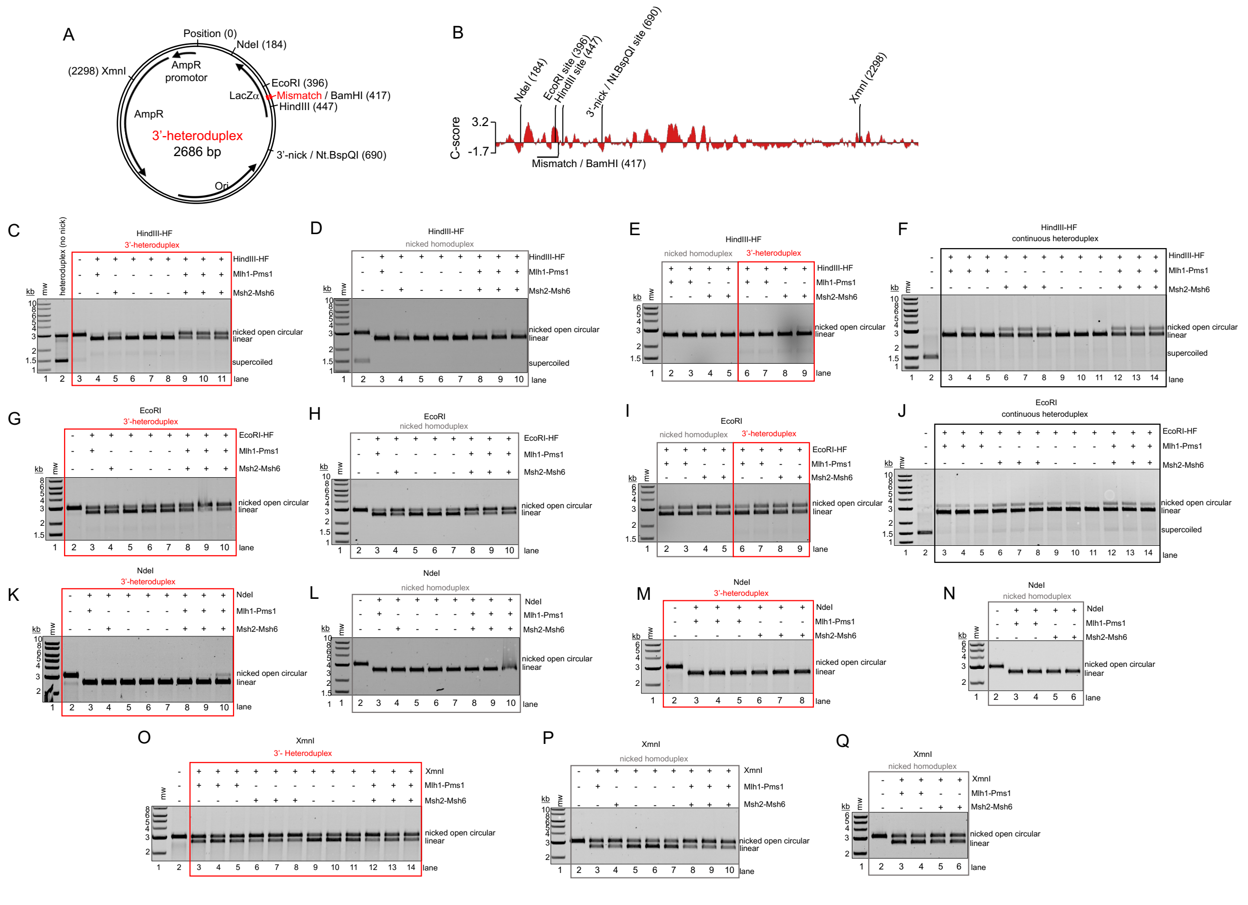

Figure S1: Restriction enzyme-based assay probing for Mlh1-Pms1 and Msh2-Msh6 localization near a mismatch. (A) Plasmid map of the 2.7-kb construct used to generate the mismatch substrate. The G:T mismatch (red dashes) was introduced at the BamHI site, positioned between the HindIII and EcoRI restriction sites. (B) Analysis of DNA sequence cyclability using DNAcycP2 to identify potential regions of local flexibility within the plasmid. Restriction sites shown in panel A are aligned along the cyclization score. (C–Q) Representative agarose gels showing the fraction of DNA digested by 0.2 units of HindIII (C–F), EcoRI (G–J), NdeI (K–N), or XmnI (O–Q), in the presence or absence of the mismatch repair proteins Mlh1-Pms1 (50 nM) and Msh2-Msh6 (50 nM). Gels containing lanes with identical enzyme and protein conditions were treated as independent replicate experiments; these data contributed to the final quantifications summarized in Figure 1.

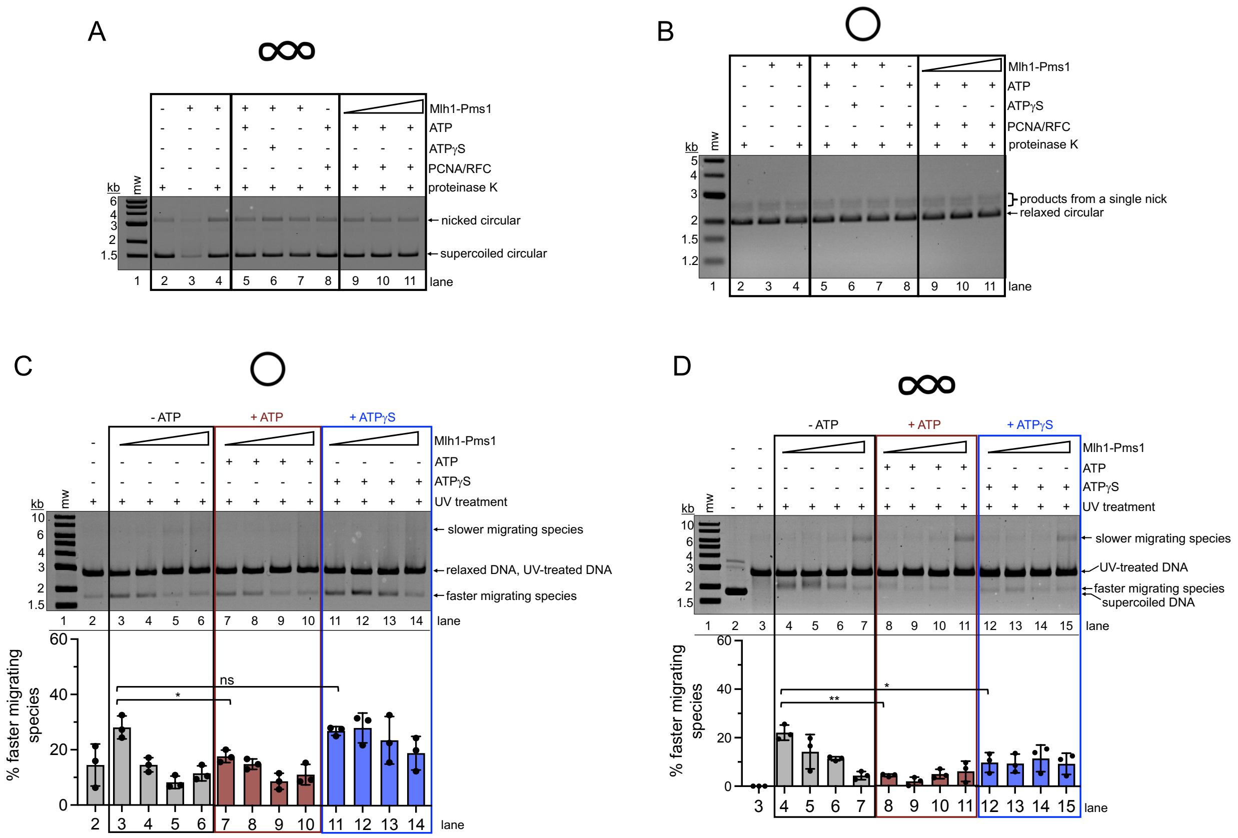

Figure S2: Endonuclease assay confirming that conditions used for UV DNA-DNA crosslinking did not permit endonuclease activity and UV-crosslinking assay at low ionic strength. (A) A supercoiled 2.7 kb pUC18 plasmid (3.8 nM) was incubated at 37ºC for 60 minutes with no UV light exposure in the same reaction conditions reported in Figure 2. Where +, Mlh1-Pms1 was included at 200 nM. Where titrated, Mlh1-Pms1 was included at 50, 100, and 200 nM, ATP was included at 0.5 mM, PCNA was included at 0.5 mM, RFC was included at 0.1 mM, and Proteinase K was 0.96 units (final concentrations). Reaction products were analyzed by a native agarose gel and endonuclease activity is measured as conversion of supercoiled circular DNA to nicked circular. Nicked products did not increase in intensity above the negative control lanes for reactions including Mlh1-Pms1 even in the presence of RFC/PCNA and ATP due to the absence of $MnSO_4$. (B) A relaxed 2.7 kb pUC18 plasmid (3.8 nM) was tested for endonuclease activity using the same conditions as in panel A. Reaction products were analyzed by an alkaline agarose gel (described in the Materials and Methods) and endonuclease activity is measured as conversion of the relaxed circular substrate to a linear single strand fragment and a closed circular single strand fragment if a single nick occurs, or degradation of the starting material if multiple nicks occur. Nicked products did not increase in intensity above the negative control lanes for reactions including Mlh1-Pms1 even in the presence of RFC/PCNA and ATP due to the absence of $MnSO_4$ on this substrate. (C) UV-based structural probing assay described in Figure 2 performed at 20 mM NaCl. Where indicated, ATP or ATPγS was 0.5 mM, and Mlh1-Pms1 was included at 50, 100, 200 and 400 nM. The amount of the faster migrating species was quantified relative to the total amount of DNA in each lane. The average of three replicates is reported. Error bars represent the standard deviation between experiments. Statistical significance was determined using unpaired t-tests with Welch's correction (Prism 10). Significance is denoted above bars (*$p < 0.05$; **$p < 0.005$; ***$p < 0.0005$; ****$p < 0.0001$; ns, not significant). (D) Identical to panel C but performed with 3.8 nM supercoiled DNA.

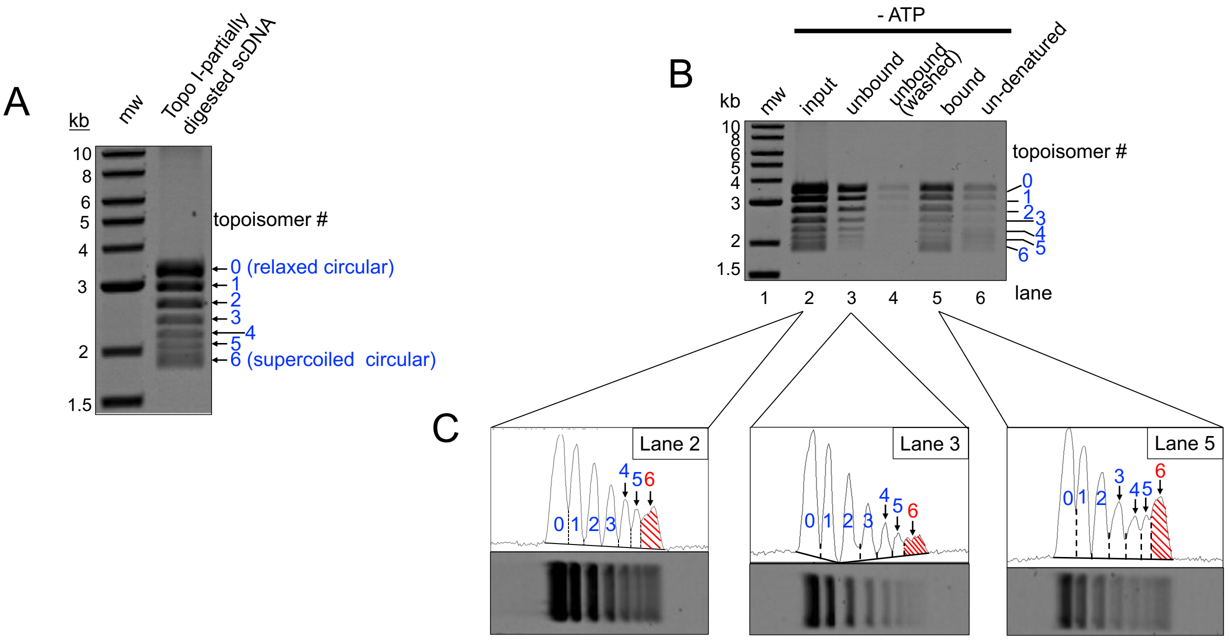

Figure S3: Quantifications for high-throughput assay measuring supercoiling density and nucleotide effects on Mlh1-Pms1 binding. (A) 2.7 kb pUC19 was partially digested with *E. coli* Topoisomerase I to generate a distribution of DNA topoisomers. Seven distinct bands are visible when analyzed by agarose gel. We arbitrarily numbered them 0 through 6, with zero being the most relaxed and six being the most supercoiled. This pool is the input for reactions in Figure 4. (B) Gel also shown in Figure 3H as an example of the assay and how quantifications were performed. (C) The quantification of topoisomer #6 is shown as an example (highlighted in red). Each lane was analyzed using ImageJ, where the bands corresponding to each topoisomer were identified (separated by dotted lines) and quantified. The total amount of each topoisomer in lanes 3–6 was confirmed to be equal to the amount present in the input (lane 2). For the bound population in lane 5, the density of each topoisomer band was calculated relative to the input (lane 2). Similarly, the amount of unbound topoisomer in lanes 3 and 4 (with lane 3 shown as an example) was calculated relative to the input. Because some topoisomer remained bound to the beads after denaturation and Proteinase K treatment (as seen in lane 6), the final data is expressed as the input amount minus the sum of the unbound fraction for each topoisomer. Note that the example peaks from ImageJ were resized to be visible here. The y-axes vary per lane.

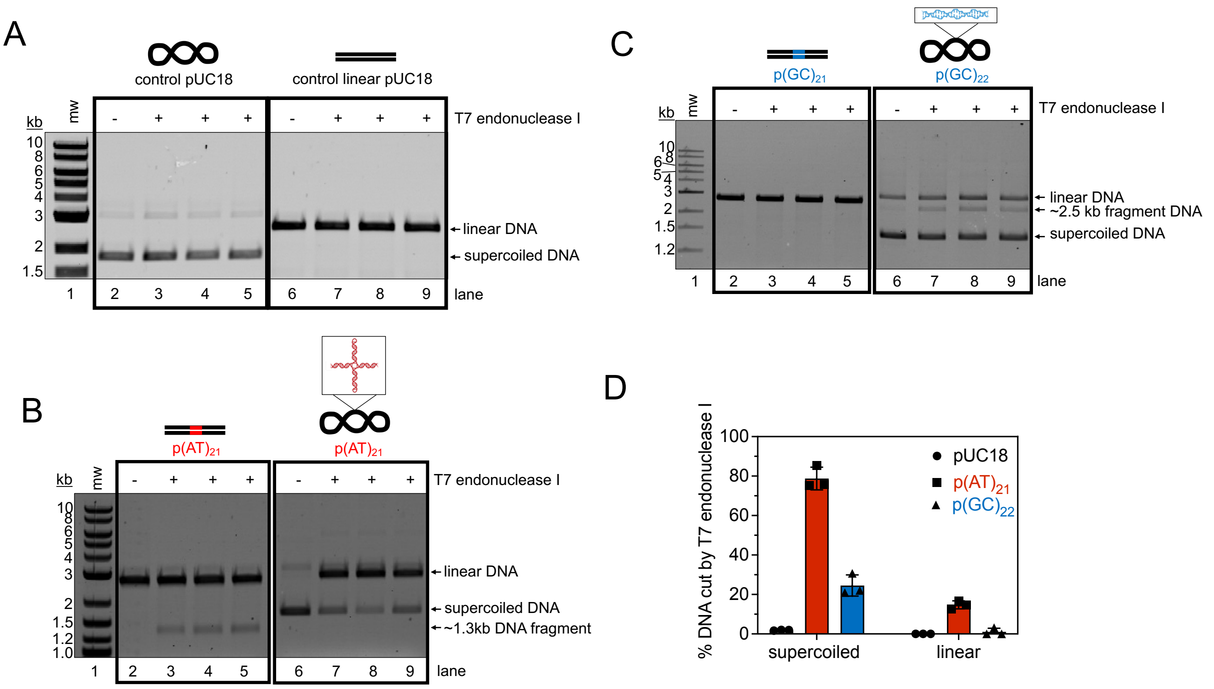

Figure S4: Characterization of plasmid substrates containing non-B-form inserts. (A-C) T7 endonuclease digests of plasmids containing the sequences described in Figure 6A. 3.8 nM of both supercoiled and linear forms of each DNA substrate were incubated with 1 unit of T7 endonuclease I and compared to reactions not treated with T7 endonuclease I. Where linear, the plasmids were linearized with BsaI-Hfv2. Lanes 3-5 and 6-9 of each are triplicate experiments (D) The average percent DNA cut by T7 endonuclease I and standard deviation for each plasmid in both supercoiled and linear forms compiled from (A-C).

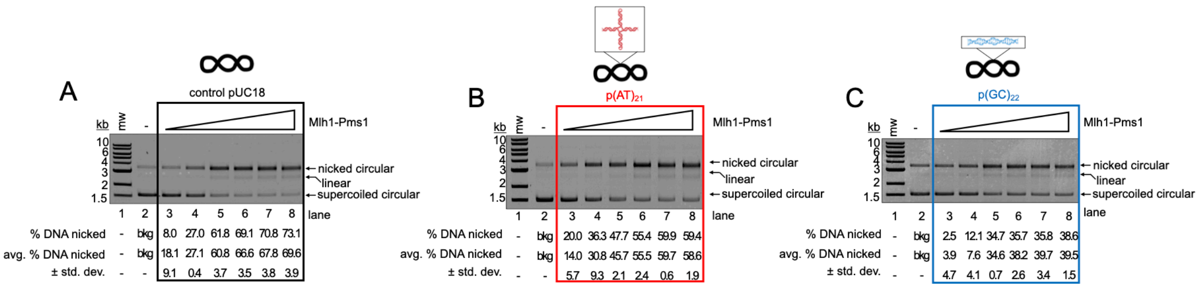

Figure S5: Endonuclease assay on supercoiled plasmids with non-B-form inserts. Endonuclease assays on substrates containing either no repeat sequence, or a $(AT)_{21}$ or $(GC)_{21}$ repeat sequence. Where indicated, Mlh1-Pms1 concentrations are 10, 25, 50, 100, 150, 200 nM. The average proportion of supercoiled DNA converted to nicked circular product from three replicates as well as the standard deviation between replicates are reported.

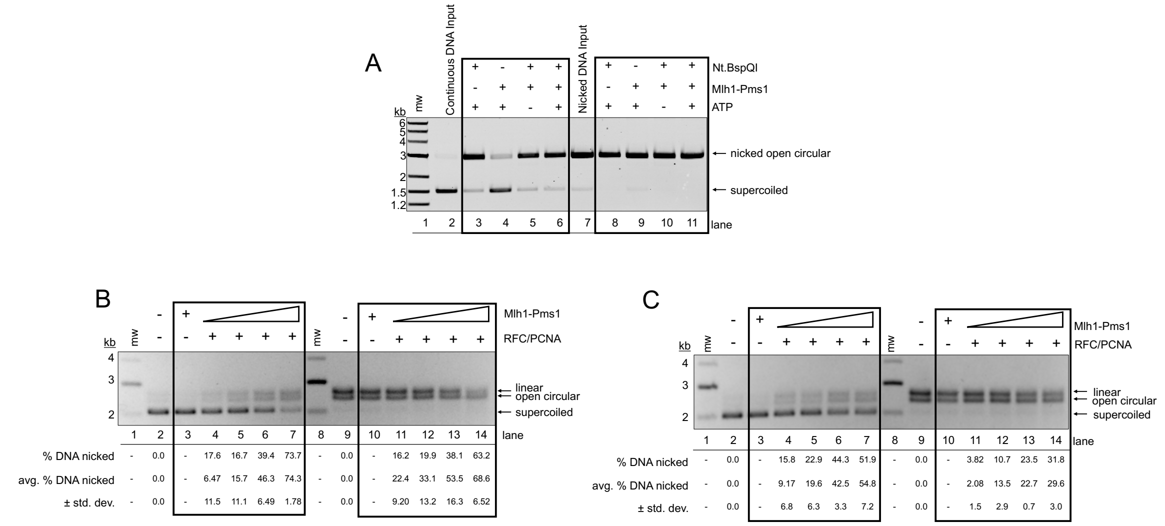

Figure S6: Mlh1-Pms1 competes with RFC for nick access to promote efficient endonuclease activity. (A) Native gel analysis of reactions from Figure 8B, demonstrating that addition of Nt.BspQI in Phase 2 efficiently nicked the DNA. (B) Representative gel from Figure 8C in which Mlh1-Pms1 was preloaded onto either supercoiled or nicked plasmids before RFC/PCNA addition. Nt.BspQI was omitted. Mlh1-Pms1 was titrated to 25, 50, 100, and 200 nM final concentrations. Other components are included as described in Figure 8B. (C) Representative gel from Figure 8C in which RFC/PCNA was incubated with either supercoiled or nicked plasmids prior to addition of Mlh1-Pms1. Except for the order of addition, reaction components and conditions were identical to those in Figure 8B, with titration points as in panel B here.
